# Essentials of Aquaphotomics and Its Chemometrics Approaches

**DOI:** 10.3389/fchem.2018.00363

**Published:** 2018-08-28

**Authors:** Roumiana Tsenkova, Jelena Munćan, Bernhard Pollner, Zoltan Kovacs

**Affiliations:** ^1^Biomeasurement Technology Laboratory, Graduate School of Agricultural Science, Kobe University, Kobe, Japan; ^2^Nanolab, Biomedical Engineering Department, Faculty of Mechanical Engineering, University of Belgrade, Belgrade, Serbia; ^3^Department for Hygiene and Medical Microbiology, Medical University of Innsbruck, Innsbruck, Austria; ^4^Department of Physics and Control, Faculty of Food Science, Szent István University, Budapest, Hungary

**Keywords:** aquaphotomics, water, near infrared spectroscopy, multivariate analysis, water spectral pattern, aquagram, aquap2

## Abstract

Aquaphotomics is a novel scientific discipline involving the study of water and aqueous systems. Using light-water interaction, it aims to extract information about the structure of water, composed of many different water molecular conformations using their absorbance bands. In aquaphotomics analysis, specific water structures (presented as water absorbance patterns) are related to their resulting functions in the aqueous systems studied, thereby building an aquaphotome—a database of water absorbance bands and patterns correlating specific water structures to their specific functions. Light-water interaction spectroscopic methods produce complex multidimensional spectral data, which require data processing and analysis to extract hidden information about the structure of water presented by its absorbance bands. The process of extracting information from water spectra in aquaphotomics requires a field–specific approach. It starts with an appropriate experimental design and execution to ensure high-quality spectral signals, followed by a multitude of spectral analysis, preprocessing and chemometrics methods to remove unwanted influences and extract water absorbance spectral pattern related to the perturbation of interest through the identification of activated water absorbance bands found among the common, consistently repeating and highly influential variables in all analytical models. The objective of this paper is to introduce the field of aquaphotomics and describe aquaphotomics multivariate analysis methodology developed during the last decade. Through a worked-out example of analysis of potassium chloride solutions supported by similar approaches from the existing aquaphotomics literature, the provided instruction should give enough information about aquaphotomics analysis i.e. to design and perform the experiment and data analysis as well as to represent water absorbance spectral pattern using various forms of aquagrams—specifically designed aquaphotomics graphs. The explained methodology is derived from analysis of near infrared spectral data of aqueous systems and will offer a useful and new tool for extracting data from informationally rich water spectra in any region. It is the hope of the authors that with this new tool at the disposal of scientists and chemometricians, pharmaceutical and biomedical spectroscopy will substantially progress beyond its state-of-the-art applications.

## Introduction to aquaphotomics

Aquaphotomics is a novel scientific discipline founded by Professor Roumiana Tsenkova at Kobe University, Japan, in 2005 (Tsenkova, 2005, 2006a,b,c, 2009) with the objective of studying and systematizing knowledge about water-light interaction, which was found to be a huge source of information on the subject of the structural and related functional properties of aqueous systems. This is a complementary “omics” discipline dealing with the large-scale, comprehensive study of water as the “*molecular and energy mirror*” of the rest of the aqueous system. While proteomics studies proteins, glycomics—carbohydrates and lipidomics—lipids; aquaphotomics explores the roles, relationships and functions of the water—an equally important biomolecule and one of nature's fundamental building blocks.

The word “aquaphotomics” is derived from the words *aqua*—water and *photo*-light since this new discipline studies water by using its interaction with the light. Thus, aquaphotomics is a science which uses water-light interaction to explore the structure of water—as a system and matrix composed of many different water molecular conformations, thereby resulting in various functionalities (Tsenkova, 2009). The main objective of establishing aquaphotomics as a novel scientific discipline was to provide a common platform and strategy to lead to an improved general understanding of the water functionality by utilizing water-light interaction at every frequency of the electromagnetic spectrum. The majority of aquaphotomics works so far have been done by using near infrared (NIR) spectroscopy, especially in the area of the 1st overtone of the OH stretching band (1,300–1,600 nm) where many water absorbance bands are identified and consistent with previously reported or calculated overtones of water absorbance bands in the infrared region (Weber et al., 2000, 2001; Smith et al., 2005; Tsenkova, 2009; Tsenkova et al., 2015). What aquaphotomics research studies showed is that NIR spectroscopy, and in general water-light interaction over the entire electromagnetic spectrum, can significantly contribute to the field of water science and better understanding of water molecular systems (Tsenkova, 2009).

The NIR wavelength region from around 680 to 2,500 nm is considered as an excellent tool for water observation that provides an enormous amount of information about water molecular structure (Büning-Pfaue, 2003; Tsenkova, 2009). The NIR light allows a longer penetration length, as compared to infrared, even up to 10 mm in the short wavelength region (750–1,100 nm) (Workman, 2000), making it a rapid and non-destructive measurement technique particularly suitable for studying intact biological systems. Numerous NIR spectra can be obtained in various conditions and states of the systems (under different perturbations)—all in real time. NIR spectroscopy has a rich history of applications in pharmaceutical and medical fields. Water, however, with its NIR characteristic spectrum was often seen as a problematic component and the common source of measurement error, because it could alter sample spectra, hide weak absorbance bands and shift other absorbance bands (Ciurczak and Igne, 2014). In fact, water is cited as one of the main disadvantages of NIR spectroscopy in pharmaceutical applications since it prevents a direct quantification (Jamrógiewicz, 2012).

Traditionally, water bands in the NIR region around 1,440 nm (the first overtone of OH stretch) and 1,940 nm (a combination of OH bending and stretching) have been very useful in the studies of the state of water in various samples (Ozaki, 2002). One of the major and most common applications of NIR spectroscopy was moisture determination (Osborne et al., 1993; Reeves, 1995). NIR spectroscopy has been used to investigate water content, hydrogen bonds and hydration state in a variety of fields such as agriculture and food industry, medical and pharmaceutical sciences, and polymer and textile industries (Ozaki, 2002).

Although some early works on water analysis reported the rich informational potential of its NIR spectrum (Hirschfeld, 1985; Iwamoto et al., 1987; Grant et al., 1989; Maeda et al., 1995), it was only with the development of aquaphotomics that the properties of water as a “collective matter and energy mirror” were truly explored (Tsenkova, 2009). The so-called “*water mirror approach”* of aquaphotomics utilizes the high sensitivity of water's hydrogen bonds, where all the components of the aqueous system and surrounding energies influence the water structure, i.e., the covalent bonds. Every aqueous system is a dynamic arrangement of water molecular network hydrogen-bonded to other constituents and influenced by perturbations. Any perturbation of the aqueous system results in changes of water molecular conformations, which in turn produce changes in the corresponding NIR spectra at their respective water absorbance bands. As a consequence of the strong potential of water molecules for hydrogen bonding, water, a natural matrix of any aqueous or biological system, changes its absorbance pattern every time it adapts to a physical or chemical change in the system itself or its environment (Tsenkova, 2008c). It is this quality of water that indirectly permits measurements of small quantities or structural changes of other molecules present in the aqueous system. By tracking the changes of water absorbance bands in the spectra of aqueous or biological systems, the information is extracted about not only water structure but also other components present in water or the state of the system as a whole (Tsenkova, 2006c, 2007, 2008b, 2009).

Being rapid and non-destructive, NIR spectroscopy is a powerful technique with an incredible range of applications, whose horizons have been further expanded by aquaphotomics. Since its establishment more than a decade ago, aquaphotomics has grown into a vast and multidisciplinary scientific field, encompassing many research areas (Table 1). Changes in the absorption spectrum of water are used for quantification of the solutes present in water, even when the solutes do not absorb NIR light at all (Grant et al., 1989; Tsenkova, 2009; Gowen et al., 2015). This so-called water-mirror approach enables measurements of concentrations previously impossible with NIR spectroscopy at ppm levels (Sakudo et al., 2006b; Tsenkova, 2008b; Gowen et al., 2013; Bázár et al., 2014, 2015), and even at ppb levels under certain experimental conditions (Sakudo et al., 2005, 2006b; Tsenkova et al., 2007b; Tsenkova, 2008a,b). Furthermore, the aquaphotomics research of biological systems introduced a concept of water spectral pattern as a holistic biomarker (Tsenkova, 2006c, 2007), which relates certain structures of water with functionalities of the respective biological systems, thus opening new directions toward non-destructive quality monitoring applications and non-invasive biodiagnosis.

**Table 1:** Fields of aquaphotomics applications.

**Application**	**References**
Fundamental biochemical studies of water solutions	Sugars (Bázár et al., 2015; Cui et al., 2017a), proteins (Tsenkova et al., 2004; Chatani et al., 2014), DNA (Goto et al., 2015), salts (Gowen et al., 2013, 2015), alkali-metal halides (Kojić et al., 2014), acids (Omar et al., 2012), and metal ions (Sakudo et al., 2006b; Tsenkova et al., 2007a; Putra et al., 2010)
Water quality	Water filtration process (Cattaneo et al., 2011), detection and quantification of pesticides (Gowen et al., 2011), discrimination of mineral waters (Munćan et al., 2014), detection of contaminants (Gowen et al., 2015), and holistic water monitoring (Kovacs et al., 2016)
Food quality	Various foodstuff (Gowen, 2012), cheese (Atanassova, 2015), honey (Bázár et al., 2016), mushrooms (Gowen et al., 2009a), bacteria in food (Nakakimura et al., 2012), milk (Tsenkova, 1994; Tsenkova et al., 2001a,b), and food packaging influence (Cattaneo et al., 2016; Barzaghi et al., 2017)
Materials and nanomaterials	Soft contact lenses (Munćan et al., 2016b; Šakota Rosić et al., 2016) fullerene based nanomaterials (Matija et al., 2012, 2017), and polystyrene particles (Tsenkova et al., 2007b)
Microbiology	Bacteria (Nakakimura et al., 2012; Remagni et al., 2013; Slavchev et al., 2015, 2017), and HIV virus (Sakudo et al., 2005)
Plant biology	Mosaic virus detection in soybeans (Jinendra et al., 2010), and abiotic and biotic stress (Jinendra, 2011)
Animal medicine	Mastitis in cows (Tsenkova et al., 2001a,b,c, 2005; Tsenkova and Atanassova, 2002; Atanassova et al., 2009; Meilina et al., 2009), udder health (Tsenkova, 1994), ovulation period in Bornean orangutan (Kinoshita et al., 2016), ovulation period in giant pandas (Kinoshita et al., 2010, 2012), estrus detection in cows (Takemura et al., 2015), and tissue discrimination (Sakudo et al., 2006a)
Human medicine	DNA mutations (Goto et al., 2015), HIV virus detection (Sakudo et al., 2005), tissue discrimination (Sakudo et al., 2006a), the state of metals in tissues (Sakudo et al., 2007), prion protein disease (Tsenkova et al., 2004), skin cream effects (Matija et al., 2013) dialysis efficacy monitoring (Munćan et al., 2016a), colorectal cancer diagnostics (Munćan et al., 2016a)

The aquaphotomics research fields have two things in common. First, water is the common matrix of all the systems studied. Second, the approach to extract the information hidden in complex and multidimensional spectra of such systems requires a specific aquaphotomics methodology developed over the years and based on rich experience in dealing with a great variety of aqueous systems. The objective of this paper is to provide guidance about how to perform aquaphotomics analysis of NIR data. Using an example dataset of aqueous salt solutions, each step of the analysis will be explained and supplemented by similar examples from the existing literature illustrating how specific steps in data analysis provide new insights, improve spectral quality, or reveal new information. The basic methodology explained in this work is applicable to the analysis of NIR data of any aqueous system, with minor aqueous system- and purpose-specific adjustments. A step-by-step explanation of aquaphotomics analysis supplemented by citations of similar works will provide a solid basic knowledge about how to start and perform the analysis as well as where to look for further information. It is the hope of the authors that, with this new tool at the disposal of scientists and chemometricians, pharmaceutical and biomedical spectroscopy will utilize the richness of NIR water spectra to extend its applications far beyond moisture determination, leading to a substantial progress beyond the current state of the art.

## Glossary of aquaphotomics terms

This glossary is intended to define the terms and certain abbreviations commonly used in the aquaphotomics literature, which will appear throughout this paper. New terminology has emerged over time and with the development of aquaphotomics and the resulting need to better describe its subject of exploration using newly discovered knowledge. The origin and definitions for the terms are compiled from several sources, which are listed in the respective columns of Table 2.

**Table 2:** Glossary of aquaphotomics terms.

**Term**	**Definition**
Water Mirror Approach (Tsenkova, 2008b, 2009)	Aquaphotomics spectral analysis is often called “water mirror approach” because of the indirect manner of acquiring information about solute composition or surroundings of the aqueous system, namely by measuring the changes in absorbance at water absorbance bands in the spectrum of the aqueous system (Tsenkova, 2009).
WAMACS - Water Matrix Absorbance Coordinates (Tsenkova, 2009)	The WAMACS are spectral ranges, where specific water absorbance bands related to specific water molecular conformations (water species, water molecular structures) are found with the highest probability (Tsenkova, 2009). For the first overtone of water (1300-1600nm), 12 WAMACs (labeled Ci, i = 1, 12) have been experimentally discovered (each 6-20nm width) and they have been confirmed by overtone calculations of already reported water bands in the infrared range (Tsenkova, 2009).
WABS – Water Absorbance Bands (Tsenkova, 2009)	Studies in the infrared range have identified the absorbance bands of numerous water species (Buijs and Choppin, 1963; Fornés and Chaussidon, 1978; Doster et al., 1986; Maeda et al., 1995; Sartor et al., 1995; Luck, 1998; Czarnik-Matusewicz et al., 1999; Heiman and Licht, 1999; Murayama et al., 2000; Segtnan et al., 2001; Chandler, 2002; Cupane et al., 2002; Šašić et al., 2002; Robertson et al., 2003). When their overtones are calculated, it is confirmed that together with already known bands, these bands occur within the whole Vis-NIR range (Tsenkova, 2005). So far, the spectral database of water absorbance bands has more than 500 bands in the area of the first, second and third overtones of water (Tsenkova, 2009; Tsenkova et al., 2015). The systematization of already identified and discovery of new water absorbance bands related to specific water species structures is one of the ongoing aquaphotomics endeavors.
Activated water bands	When a certain perturbation of interest is shown to produce the changes at specific water absorbance bands, and when this is determined consistently and repeatedly throughout the aquaphotomics analysis, these water absorbance bands are considered “activated” by the respective perturbation.
WASP–Water Absorbance Spectral Pattern (Tsenkova, 2009)	The combination of the *activated water bands* caused by a certain perturbation defines water absorbance spectral pattern, which describes the condition of the whole aqueous system. WASP can contain huge amounts of chemical and physical information about the respective aqueous system and can be thought of as a holistic marker because it captures the structure and dynamics of the respective system as a whole. At the moment, even without the assignment and understanding of water absorbance bands, WASPs can be used as holistic (bio) markers for system functionality.
Aquagrams (Tsenkova, 2010)	An aquagram is a novel graphical representation of data, invented to present in a succinct manner a water absorbance spectral pattern – WASP (Tsenkova, 2010).
Aquaphotomes (Tsenkova, 2009)	An aquaphotome is the entire complement of water molecular structures produced by aqueous or biological systems in different conditions. It can be defined as a comprehensive database of all water spectral patterns with the interpretation of their functionality given a particular set of conditions of the respective system, (Tsenkova, 2009). Every aquaphotome is system-specific. Once a large database of characteristic water bands has been acquired, they can be related to specific biological functions and subsequently used for prediction, diagnosis, and understanding of biology, chemistry and physics of biological and aqueous systems (Tsenkova, 2009).

With the main terms explained, we can now formulate the objective of aquaphotomics analysis i.e., the water mirror approach to analyze aqueous systems as a whole, using their multidimensional spectra and focusing on water absorbance bands located at specific regions, allows observation and absorbance measurements. When activated water absorbance bands are found in response to some perturbation of interest, then a water absorbance spectral pattern caused by the respective perturbation is identified. By compiling water absorbance patterns in an aquaphotome, aquaphotomics builds up a comprehensive database of the states of the analyzed system as a whole, in terms of identified water structures shaped by various internal or external perturbations. In future applications, aquaphotome database will provide a rapid identification of causes for changes and influences on the system based on the recognized water spectral patterns, which serve as holistic markers of the state of the aqueous system or biomarkers in the case of biological systems (Tsenkova, 2006c; Kovacs et al., 2016).

## Aquaphotomics methods

### Basic workflow and general guidance

**Figure 1:**
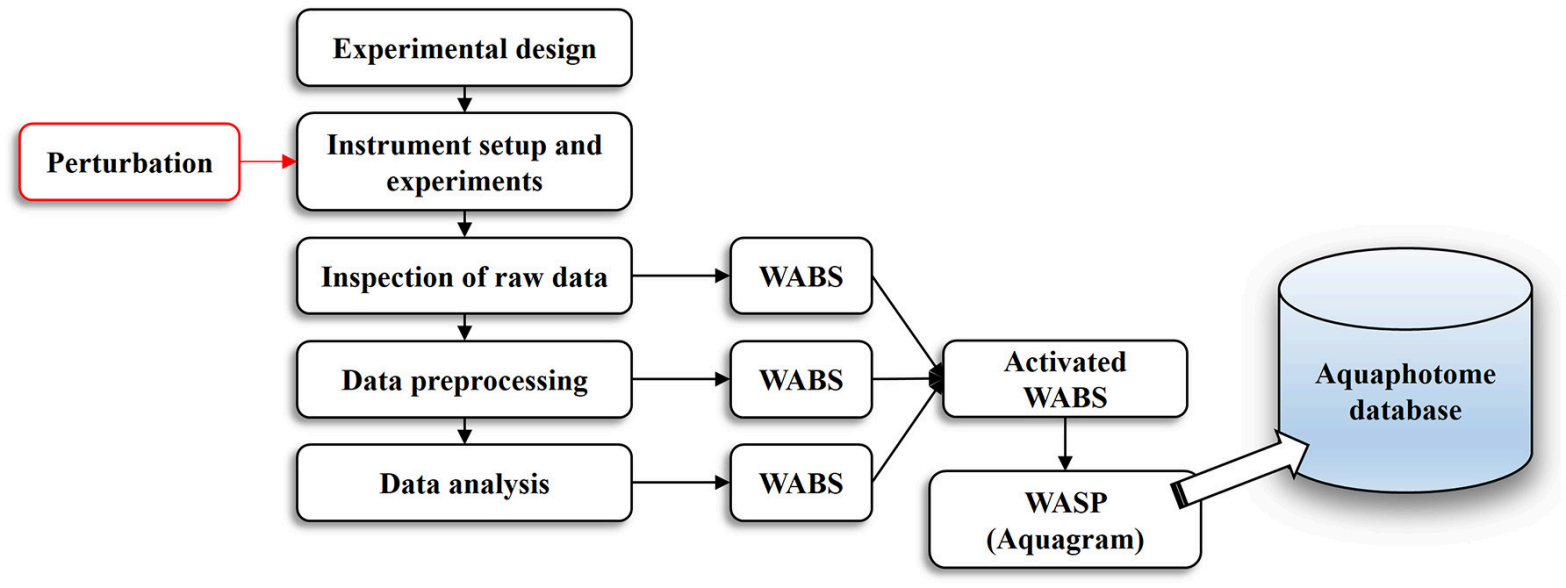
An overview of the aquaphotomics basic methodology for design, performance and analysis of experimental data with the aim of extracting water spectral pattern for the defined perturbation.

The basic workflow of aquaphotomics analysis from the experimental design to the final act of building an aquaphotome is illustrated in Figure 1. Similar to every conventional NIR spectroscopy work, everything starts with a proper experimental design and instrumental setup.

Although NIR spectroscopy, in general, does not require sample preparation, there are some specific aspects in aquaphotomics experimental design requiring more attention.

First of all, it is an absolute must to ensure that the instruments have high-quality spectral signals. In general, not all spectrometer systems are suited for aquaphotomics experiments. It is advisable to check the instrument's performance beforehand to ensure the high quality of the spectra in the entire Vis-NIR region (400–2,500 nm). All subsequent analysis will be highly influenced by the quality of raw spectral data. It is therefore of the utmost importance to evaluate raw spectra prior to any real experimental work. The basic analytical procedures for detecting errors of NIR data and evaluation of signal quality have been recently provided in an extensive study performed by Bazar et al., which tested and compared the performance of three spectrometer systems (Bazar et al., 2016). This paper can be used as a general guidance on how to test the quality and performance of NIR instrument before venturing further.

Ensuring good spectral quality is particularly important since, in addition to the already known complexity of NIR spectra due to the overtone and combination modes resulting in broad bands, the changes in the spectra of aqueous systems caused by some perturbation of interest are small and subtle. The useful information may end up being buried in noise if the instrument does not provide a high signal-to-noise ratio. Another prerequisite is the use of a high-resolution instrument. Water absorbance bands in the NIR range are usually located very close to each other, so high spectral resolution of 0.5 or 1 nm will ensure an optimal detection and separation of the bands in a subsequent analysis.

An experiment should be carried out according to previously defined protocols to ensure the same environmental conditions. The purpose of carefully designed and established protocols is to minimize the influence of unknown factors that may affect sample spectra.

The specificity of experimental design may vary depending on the type of aqueous system involved; however, the design must ensure that each sample is presented with several replicates (sample replicates) and each measurement is performed by using several consecutive illuminations (consecutive replicates, consecutive spectra). Collecting and averaging multiple scans is part of the standard practice to remove noise—recoding 64 or more scans per one spectrum reduces the noise levels significantly (Manley, 2014). Measuring liquid samples should always start with pure water (18.2 MΩ·cm) and all subsequent measurements should be done with a cuvette always placed in the same position (the same side). The same cuvette should be used throughout the experiment. It should be first rinsed at least in triplicate with sample before final filling. After that, it is placed in the sample holder and allowed to equilibrate before scanning in order to minimize inter-sample variation.

Reference measurement (blank air) should be done before each sample measurement. The order of sample measurement and sample replicates should be completely randomized; but pure water should be always scanned after a previously defined number of samples (e.g., every 5, 7, or 10 sample measurements). There are two reasons for measurements of pure water in between samples. First, these spectra are used as an environmental control, monitoring known and unknown influences on water and could later be used to correct or remove unwanted influences from sample spectra. Second, it builds a large library of pure water spectra. There are many advantages of building such a library—it contains the spectra of pure water under various changing conditions over a longer period of time under different temperatures, humidity conditions and various day-to-day variations of the instrument and working environment. Building such a database has been proved very useful for correction in general NIR applications (Tillmann and Paul, 1998). In addition, a novel method for enhancement of spectral signals has been recently developed, which also relies on building a similar library (Kojić et al., 2017).

It is also advisable to monitor and log major external influences such as laboratory temperature, atmospheric pressure and humidity, as well as sample holder temperature or cuvette. Measuring and logging external parameters can be very useful for identification of major sources of spectral variation as well as for exploration of the dynamics of different aqueous systems under the same environmental perturbations.

As opposed to traditional NIR spectroscopy, which places emphasis on the control of the environment during the measurements, “perturbation” is often used in aquaphotomics and is sometimes even a necessary component of experiments, which helps in revealing hidden information. The analysis of aqueous systems' spectra under the influence of some chosen, intentional, perturbation can be defined as an evaluation of the system by applying changes to the selected parameters and re-estimation of the results (Tsenkova, 2007). In practice, the most frequently used perturbations to induce changes in the respective systems are changes in temperature (Gowen et al., 2013; Chatani et al., 2014; Putra et al., 2017; Wenz, 2018), consecutive illuminations (Tsenkova, 2005; Chatani et al., 2014; Wenz, 2018), and changes in dilution (Gowen et al., 2013; Wenz, 2018). Other types of perturbations can also be used to test the robustness of the models developed. Besides temperature perturbation, for example Putra et al. (2017) and Meilina et al. (2011) introduced perturbations by different metal ions to test the regression model developed for the measurement of cadmium concentrations in aqueous solutions. The use of intentional, artificially created perturbations provides a change in entropy and leads to the revelation of hidden spectral information (Tsenkova, 2006c). A recent work by Wentz on water in model membranes employed four types of perturbation in the same work in order to probe and thoroughly examine changes in the water matrix [i.e., temperature, consecutive illuminations, concentration (dilution)], and difference in molecular structure of phospholipids (fourteen identical carbon acyl chains but with polar heads differing in the presence of an hydroxyl or a choline group) (Wenz, 2018). The most frequently used intentional perturbations (consecutive illuminations or increasing temperature) result in similar changes in water matrix—an increase in the number of free water molecules, which are then available for “scanning” of the rest of the system; in other words—to interact with its components, which results in changes in sample spectra and provision of additional information. Regarding unintentional perturbations, it is always advisable to investigate what perturbations (i.e., factors) have an influence on the developed models. These perturbations may include individual differences or the presence of disease in the case of biological systems studied, or even sample thickness (Tsenkova, 2004).

The first step of analysis begins with the inspection of raw spectral data. Although NIR spectra of aqueous systems are comprised of broad, overlapping spectral bands, visual spectral inspection still remains a vital step before any further data analysis. Visual inspection gives the first clues about the presence of outliers, helps in deciding what preprocessing steps to proceed with, gains a general insight into how samples are grouped and on what spectral regions to focus the attention. All the subsequent steps—data preprocessing, conventional spectral analysis and chemometrics application, which will be described in more detail later-serve to extract the information of interest. From the aspect of conventional data analysis—with building, testing and validation of a model—either qualitative or quantitative, depending on the objective of the experiment, the work is done when suitable prediction accuracy is achieved. However, this is only half of the work done in an aquaphotomics analysis. Each step of the analysis—raw data inspection, preprocessing, conventional and chemometrics analysis (an array of exploratory, classification and regression analysis)—provide certain quantitative outputs like derivatives, subtracted spectra, regression vectors or loading vectors, discriminating power and others, which all unravel water absorbance bands most affected by perturbation of interest (WABS, Figure 1).

The NIR spectra of aqueous systems are very complex, and changes in their absorbance spectra caused by some perturbation will usually be very subtle, but nonetheless persistent and consistent. From all the WABs discovered during multiple steps of aquaphotomics analysis, a noticeable pattern of repeating, common absorbance bands will emerge to reveal perturbation-induced water absorbance bands i.e., how and what water molecular conformations are affected. When this absorbance spectral pattern water absorbance pattern (WASP) is recognized, it can be presented in a simple, yet concise and informative manner by using aquagrams. This aspect of aquaphotomics analysis adds one more dimension to the results obtained in that it provides understanding of the water functionality in the respective system. It allows linking discovered WASPs with the conditions of the aqueous systems analyzed, revealing how and why water changes the way it does under certain perturbation. This is of special importance for living, biological systems. The storing of WASPs into a large aquaphotome database allows for a fast comparison and identification of the state of aqueous or biological systems, thereby in essence providing biodiagnosis based on the state of water.

### Aquaphotomics analysis of potassium chloride solutions—a worked-out example

To better illustrate the working process of aquaphotomics analysis, we will present an example of analysis performed on the spectral dataset of aqueous solutions of potassium chloride in the next sessions. The perturbation of the water matrix by salt and measurement of salt concentration are already available in aquaphotomics literature (Gowen et al., 2015) and even in very early near infrared spectroscopy applications (Grant et al., 1989). We have chosen this perturbation since it perfectly illustrates the aquaphotomics water-molecular and energy mirror concept in that the salts are practically transparent for NIR light. Therefore, the results obtained thereby are based entirely on the changes in the water molecular matrix. Experimental condition will be described next.

#### Materials and methods

##### Sample preparation

Potassium-chloride (KCl, M = 74.56 g.mol^−1^, purity ≥ 99.0% w/w, Wako Pure Chemical Industries, Ltd. Kobe, Japan) was used.

All samples were prepared by using deionized water from a Milli-Q water purification system (Millipore, Molsheim, France). A stock solution of 100 mM was prepared at first. Working solutions were made by serial dilution of the stock solution in 10-mM steps to produce the following KCl concentrations: 10, 20, 30, 40, 50, 60, 70, 80, and 90 mM. All samples of the stock and working solutions were freshly prepared in two independent sample replicates (i.e. a total of 20 samples for the analysis).

##### NIR spectra collection

Transmittance spectra of KCl aqueous solutions were acquired by using a FOSS-XDS spectrometer (FOSS NIRSystems, Inc., Hoganas, Sweden) equipped with a Rapid Liquid Analyzer module consisting of a temperature-controlled cuvette holder. The temperature of the sample holder was kept constant at 28°C during all measurements. This temperature was chosen to be close to the ambient temperature (ca. 28°C), allowing a fast and easy way of maintaining constant temperature during measurements. Each sample was firstly incubated in the sample holder for 90 s before scanning to get the required temperature of 28°C. Deionized water samples were measured as an environmental control for every five sample measurements. Spectral acquisition order was randomized with respect to salt concentration. The 1-mm path length quartz sample cell was used as a container.

The spectra were acquired in the range of 400–2,500 nm, with a resolution of 0.5 nm. Each saved spectrum was an average of 32 successive scans. This number of scans was chosen to shorten the acquisition time. Three consecutive spectra were recorded for each sample and for each measurement. The reference spectrum was recorded before each measurement. The spectral data were transformed to pseudo-absorbance units (logT^−1^, where *T* = transmittance). One sample was represented by six spectra in total, from two independent sample replicates and three consecutive spectra. The total number of recorded spectra was 75 (10 concentrations × 2 sample replicates × 3 consecutive scans + 15 control scans of deionized water).

The FOSS-XDS instrument was operated by using VISION 3.5 software (FOSS NIRSystems, Inc., Hoganas, Sweden).

##### Data analysis

For the purpose of this paper, the data analysis of KCl solutions was performed by using only the wavelength range from 1,300 to 1,600 nm, which represents the absorption region of OH bonds of water (1st overtone of OH).

Smoothed spectra were calculated by using a Savitzky-Golay polynomial filter (2nd order polynomial fit and 21 points). Difference spectra were calculated by subtraction the average spectrum of deionized water from the average spectra of potassium-chloride solutions for each concentration level. The 2nd derivative spectra of potassium-chloride solutions were calculated by using a Savitzky-Golay filter (2nd order polynomial fit and 21 points). Principal component analysis (PCA) was used to describe multidimensional patterns in the spectral data and to discover outliers. The relationship between the actual and predicted concentrations of KCl was examined by using Partial Least Squares Regression (PLSR) based on leave-one (concentration)-out cross validation, i.e., without six spectra of the two independent sample replicates at a time during the iterative validation process.

The regression was performed on the previously smoothed (Savitzky-Golay filter, 2nd order polynomial filter, 21 points) and multiplicative scatter corrected (MSC) spectra in the spectral range of 1,300–1,600 nm. The precision and accuracy of the developed PLSR model were evaluated by the coefficient of determination (*R*^2^) and root mean square error (RMSE) of cross-validation.

Raw spectra, difference spectra, loading vectors of PCA analysis, and regression vector of PLSR analysis were examined in order to find and assign characteristic water absorbance bands showing considerable changes in response to changes in KCl concentration. Thus, identified bands were used to describe water spectral pattern of salt solutions. To visually represent changes of water spectral pattern as a function of salt concentration, different types of aquagrams were constructed, namely classic aquagrams, aquagrams with confidence intervals and temperature-based aquagrams. The instructions for all necessary calculations and steps to produce these charts are explained in a separate section (Water spectral pattern represented by aquagrams).

All data analysis was performed by using R Project for Statistical Computing (R Core Team, 2017) (RRID:SCR_001905) and an “aquap2” package (Pollner and Kovacs, 2016).

### Aquap2 package

The “aquap2” package developed by Pollner and Kovacs (2016) (free download and instructions available at www.aquaphotomics.com) provides an easy-to-use data preparation and analysis tools developed for extending the functionalities of the R project software to the needs of aquaphotomics. It is a non-commercial, free-to-use software, which can dramatically speed up analysis time, especially in the case of large datasets. It is very flexible and allows an automation of highly repetitive tasks, while also providing special functionalities not available in other commercially available chemometrics software, such as frequently used graph—aquagrams.

Aquap2 package offers the following functionalities:

- Experimental design with randomization of samples, planned number of replicates, consecutives, and environmental control samples
- Data import from various file formats suited for a variety of spectral acquisition softwares
- Fusion of spectral data with data from data loggers monitoring the environment or sample holders
- Flexible data analysis customized for different grouping / splitting / slicing of data with encapsulated, i.e., stable color-coding of samples/groups
- Very flexible data visualization from raw spectra to automatically detected and labeled peaks in various multivariate models' outputs
- A variety of data pre-treatments (e.g., smoothing, standard normal variate transformation (SNV), multiplicative scatter correction (MSC), extended multiplicative scatter correction (EMSC), detrend transformation, derivatives (using different methods), averaging, resampling, artificial noise loading
- Chemometrics methods: principal component analysis (PCA), partial least squares regression (PLSR), soft independent modeling of class analogies (SIMCA) and different versions of aquagrams
- Different cross-validation and independent prediction options to support model optimization

## The power of raw spectra and conventional spectroscopic analysis

With so many chemometrics methods available, one often neglects the possibility that something can be extracted from the raw spectra, especially since changes in the water spectra in the near infrared region are subtle and difficult to observe with the naked eyes. However, the first, most natural step in all data analysis is to inspect the raw data.

In the NIR region, the water spectrum consists of four main maxima located approximately at 970, 1,190, 1,450, and 1,940 nm, which are due to the second overtone of the OH stretching band (3ν_1,3_), combination of the first overtone of the OH stretching and OH bending band (2ν_1,3_ + ν_2_), the first overtone of the OH stretching band (2ν_1,3_) and combination of the OH stretching and OH bending band (2ν_1,3_+ ν_2_), respectively (Luck, 1974). All these regions are informationally valuable. So far, more than 500 water absorbance bands have been identified under these broad peaks (Tsenkova, 2009; Tsenkova et al., 2015). Depending on the type of aqueous system, some regions can prove to be more suitable for analysis and provide more information; hence it is always advisable to closely examine each of these regions.

**Figure 2:**
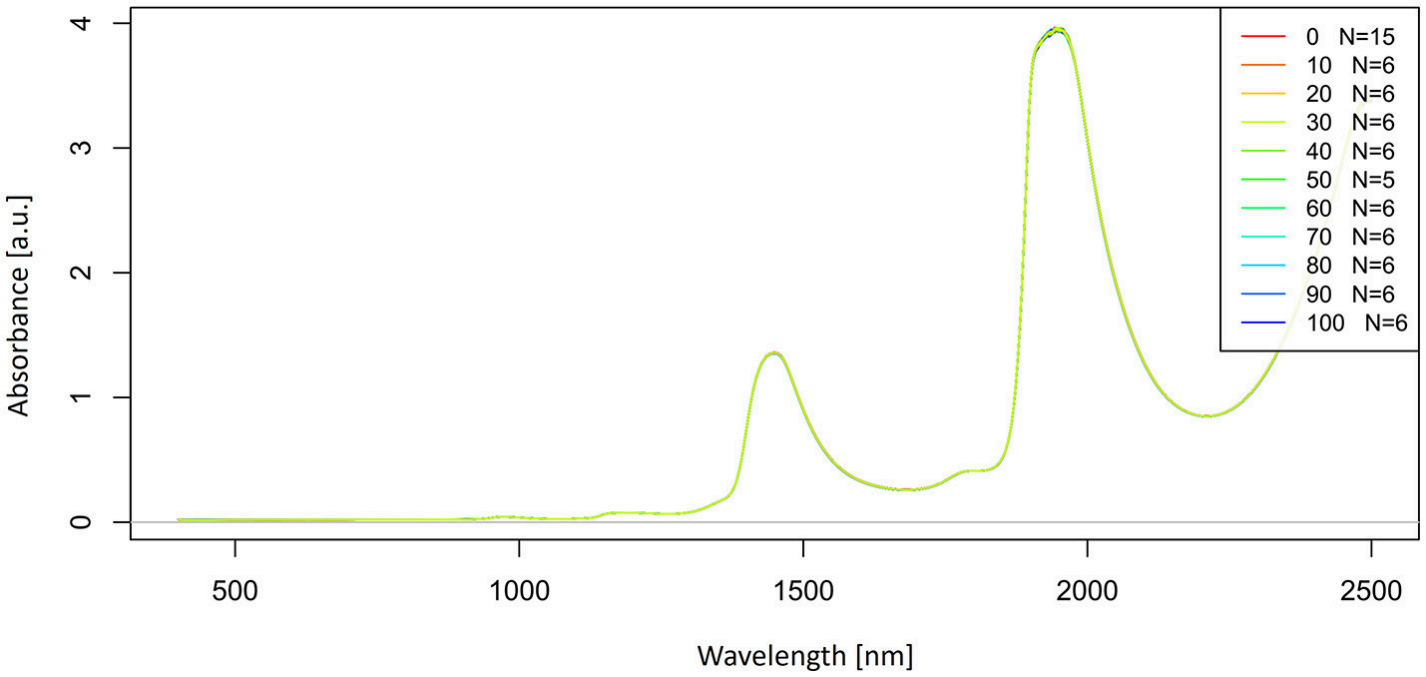
Raw absorbance (logT-1) spectra in the entire spectral range of Milli-Q water and aqueous solutions of potassium-chloride in the concentration range of 10–100 mM.

Let us now look at the raw, untreated spectra acquired for our potassium chloride example dataset (Figure 2).

The raw spectra were plotted to visualize the spectral changes introduced by adding different concentrations of salt to pure water. Two large peaks (around 1,450 and 1,940 nm attributed to the first overtone and combination region of OH stretching and bending vibrations) dominate the spectra of potassium chloride solutions. It is logical because salts do not exhibit the NIR spectra. Very small, broad features can also be observed around 1,190 nm. The region of the combination band shows significant noise due to the high absorption of water, which far exceeds 3 absorbance units and will be excluded from subsequent analysis. Further analysis will be performed only in the region of the first overtone of water, where for the most part, water absorbance bands can be clearly resolved and for which good literature sources exist about the specific assignments of water molecular conformations.

In this stage of data evaluation, two types of calculations are usually performed: averaging and spectral subtraction. The averaging can be done across all spectral consecutives and sample replicates. At this stage, the goal of averaging is to eliminate the influence of variations, which are not of primary interest, such as those attributable to different temperatures, humidity, or consecutive illumination. The average spectra of different groups of samples calculated this way will better reveal differences among different sample groups. However, the averaged spectra are influenced by outliers, so some measures of detecting and eliminating them should be taken before this step.

The next step is a spectral subtraction, which produces difference spectra. This is a very effective way for detection of subtle differences between the two spectra (Ozaki et al., 2003).

There are many approaches to spectral subtraction, and the simplest, classical approach is to subtract from the average spectrum of all samples, the averaged spectrum of pure water measured as a control during the experiment or of the solvent. This is the most simple and efficient method of bringing immediately a better visualization and observation of the water bands hidden under broad overtone and combination peaks.

Another subtraction method, recently developed, proposes a “closest spectrum” subtraction (Kojić et al., 2017). This subtraction method involves creating all the possible pairs of differences (solution—pure solvent) and finding the closest spectral pair (minimal difference) based on the smallest area under the curve of the difference spectrum. Thus, the found spectrum, the “closest spectrum,” is then subtracted from the remaining spectra. Pure solvent spectra can be acquired during the experiment or found in a library of solvent spectra which must be previously created by performing an acquisition under various, mainly temperature, perturbations. This method provides, on average, a 4-fold increase in precision as compared to traditionally used average spectrum subtraction (Kojić et al., 2017).

Another way of enhancing differences is to calculate the difference spectrum along some perturbation of interest. This type of subtraction can reveal water absorbance bands activated by a particular perturbation. This simple approach, for example, allowed an immediate identification of main differences in the water structure between the groups of bacterial cultures *S. auerus* and *E. coli* (Nakakimura et al., 2012). In addition, in the study of the effect of soybean mosaic virus, the difference spectrum between the average spectra of healthy and diseased plants clearly revealed water absorbance bands due to virus-induced changes (Jinendra et al., 2010). Another example can be found in a study of the spectral behavior of mushrooms subjected to physical perturbation by different levels of mechanical vibration (Gowen et al., 2009b). The difference spectra obtained by subtracting the averaged spectrum of undamaged mushrooms from averaged spectra of damaged mushrooms subjected to different perturbation levels revealed sharp features around 1,398 nm for the two highest level of perturbations, which corresponds to absorption of free single water molecules trapped by ions (Kojić et al., 2014) at the mushroom surface originated from physically damaged cell walls.

Another highly efficient approach in revealing different water dynamics in samples is a subtraction of the 1st consecutive spectra from all other consecutive measurements. This subtraction technique was first applied in a study of different prion protein isoforms in water solutions (Tsenkova et al., 2004; Tsenkova, 2005), when it was shown for the first time that illumination changes the water system and each consecutive spectrum of the sample is influenced by light absorption. The effect of absorbed photons on water molecular systems increased a number of free water molecules available to interact with solutes in the aqueous system, performing “scanning” of solutes and the rest of the water molecular system resulting in changes of the corresponding spectra. In this way, additional information can be extracted, which is especially beneficial when the aqueous systems analyzed are very similar. In the case of the prion protein study, this approach revealed drastic differences in the free O-H absorbance bands and superoxides for different prion protein isoforms (Tsenkova et al., 2004; Tsenkova, 2005).

The spectra transformed as just described can also be further analyzed by using other data-mining approaches.

## Spectral preprocessing—improving and enhancing spectral information

The fundamental problem, not only in aquaphotomics analysis but also generally in all spectral analysis, is how to extract the useful information hidden in the complex spectral measurements. The objective of preprocessing is to enhance the information of interest, and decrease or remove unwanted influences on spectral signals.

The spectral preprocessing methods include mathematical pretreatments, such as centering and normalization (mean-centering, standard normal variate transformation (SNV)(Barnes et al., 1993); noise-reduction methods, such as smoothing or wavelet transform (Patil, 2015); baseline correction methods which include de-trending (Barnes et al., 1989); multiplicative scatter correction (MSC) (Dhanoa et al., 1994); extended multiplicative scatter correction (EMSC) (Martens and Martens, 2001); and spectral derivatives which, in addition to baseline correction, also resolve overlapping peaks.

Spectral patterns collected are usually affected by noise or instrumental variations that may have a detrimental effect on further analysis and conclusions that may be drawn (Gowen and Amigo, 2012). The weakly absorbing bands in the NIR region are far more affected as compared to the stronger ones. The best approach in ensuring high-quality and noiseless spectra, begins with the conditions of spectral collection which should be carefully controlled. Usually, collecting and averaging multiple scans successfully reduce the noise. However, some level of noise should be expected so that the common practice is to use smoothing techniques (Manley, 2014).

The most common de-noising techniques used in aquaphotomics methods are based on the Savitzky–Golay approach (Savitzky and Golay, 1964), which fits the spectral pattern to a polynomial function (second-order polynomial) in a step-wise manner. Continuous wavelet transform (CWT) is also one of the de-noising techniques, proved to be very efficient for processing analytical signals (Shao et al., 2003), and is of recently frequently used for enhancing spectral resolution and background removal in aquaphotomics works (Shao et al., 2010; Kang et al., 2011; Shan et al., 2015; Cui et al., 2016).

Mean centering of spectra is a pre-processing technique mostly used with principal component analysis (Agelet and Hurburgh Jr, 2010). It involves a subtraction of the average spectrum from the entire dataset, which results in reduced number of variables and complexity of subsequently built models (Manley, 2014).

Apart from random noises, the spectra of aqueous systems often exhibit baseline variations (in slope and offset) due to the scattering originated from differences in sample surface or particle size variations (Ozaki et al., 2003). Baseline offset problems are commonly solved by the application of SNV or MSC corrections methods. MSC is a better choice for correction when variations in the spectral slope are also present as a result of additive variation, which increases with wavelength due to the scattering present in samples. The disadvantage of MSC transformation is that it is sample-dependent; hence any change in the sample set requires a recalculation of all MSC related subsequent calculations (Dhanoa et al., 1994).

Detrending is also a possible choice for correction of baseline shift and curvilinearity. This method consists of modeling the baseline as a function of wavelength with a second-degree polynomial and a subsequent subtraction of this function from each spectrum individually.

With correction for baseline variations, one should be careful as sometimes they can contain information of interest. For example, in a study of prion protein isoforms, the benefit of multiplicative scatter correction was 2-fold. First, it confirmed the presence of scattering for one isoform of prion protein, which helped better understanding of its interaction with water by explaining that an increase in bulk water and changes in protein structure are the cause of scattering. Second, when correction for the scattering was applied, a subsequent analysis revealed differences in different protein isoforms not related to the scatter (Tsenkova et al., 2004). However, in a problem of somatic cell count determination, removal of the baseline variation by application of the second derivative transformation led to a diminished accuracy of prediction of somatic cell count in milk, leading to the conclusion that the baseline correction removed significant information (Tsenkova et al., 2001a).

The use of derivation as a pre-processing technique for NIR data is quite common. There are two ways of calculating derivatives: the Norris–Williams derivation (Norris and Williams, 1984) and Savitzky–Golay derivation (Savitzky and Golay, 1964). Derivatives can solve two basic problems with NIR spectra of aqueous systems: overlapping peaks and large baseline variations. The effect of derivatives is most clearly seen in the second derivative of a spectrum, which is able to separate overlapping bands. The second effect of the second derivative is removal of baseline shifts (Williams and Norris, 1987; Heise and Winzen, 2002). Two side effects of the derivatives are the loss of the original shape of a spectral curve, which may result in a difficult data interpretation and a reduction in signal-to-noise ratio. Choosing window size when performing derivatives should also be done with caution in the case of spectra of aqueous systems because this parameter influences a number of points in the resulting spectral vector (Rinnan et al., 2009), which may lead to a wavelength loss and a subsequent loss of information about some water bands.

Iwamoto et al. (1987) showed that the derivative transformation of spectra was a useful method of separating multiple absorptions in broad spectral peaks of water and used it successfully to better understand the state of water in foodstuffs. In aquaphotomics applications, the second derivative is a very popular and efficient approach for discovering activated water absorbance bands that are not visible in the original spectrum (see for example Jinendra et al., 2010; Jinendra, 2011; Kinoshita et al., 2012; Bázár et al., 2016; Kovacs et al., 2016).

**Figure 3:**
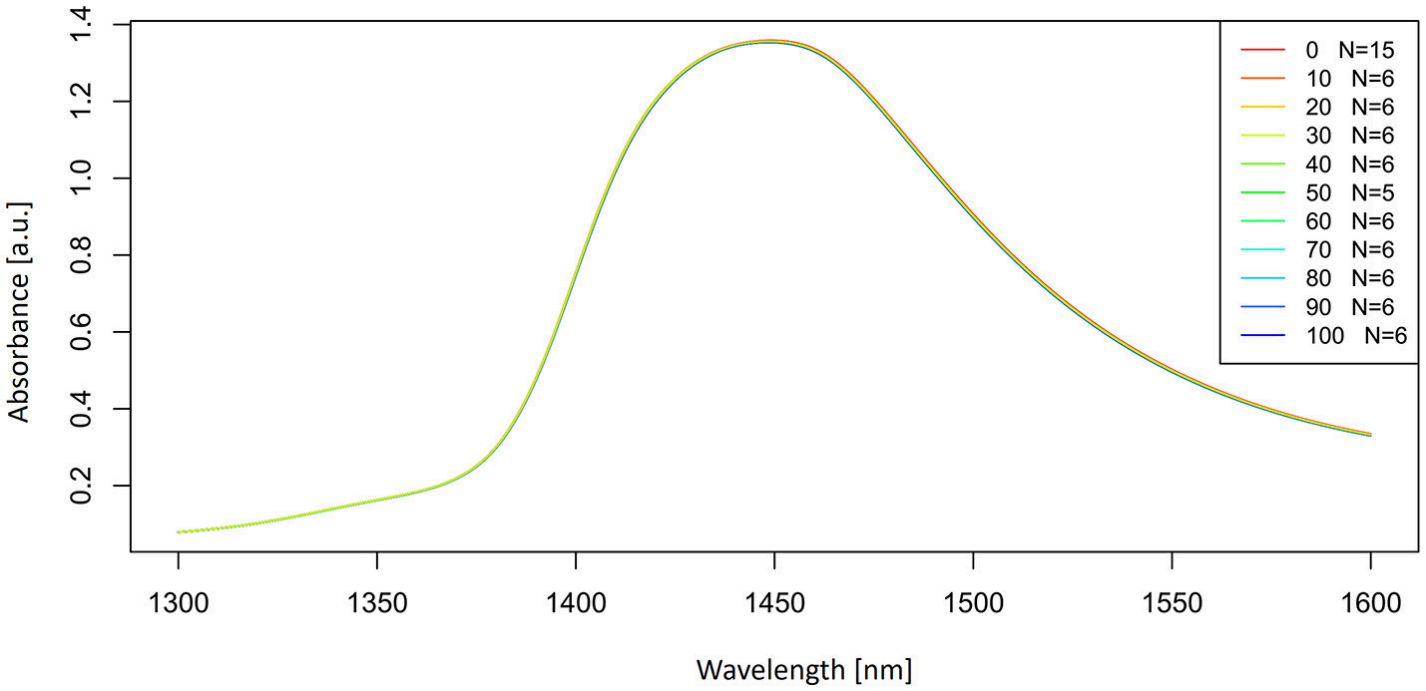
Smoothed (calculated with a Savitzky-Golay filter using 21 points) absorbance (logT-1) spectra in the spectral range of 1,300–1,600 nm (OH first overtone) of Milli-Q water and aqueous solutions of potassium-chloride in the concentration range of 10–100 mM.

**Figure 4:**
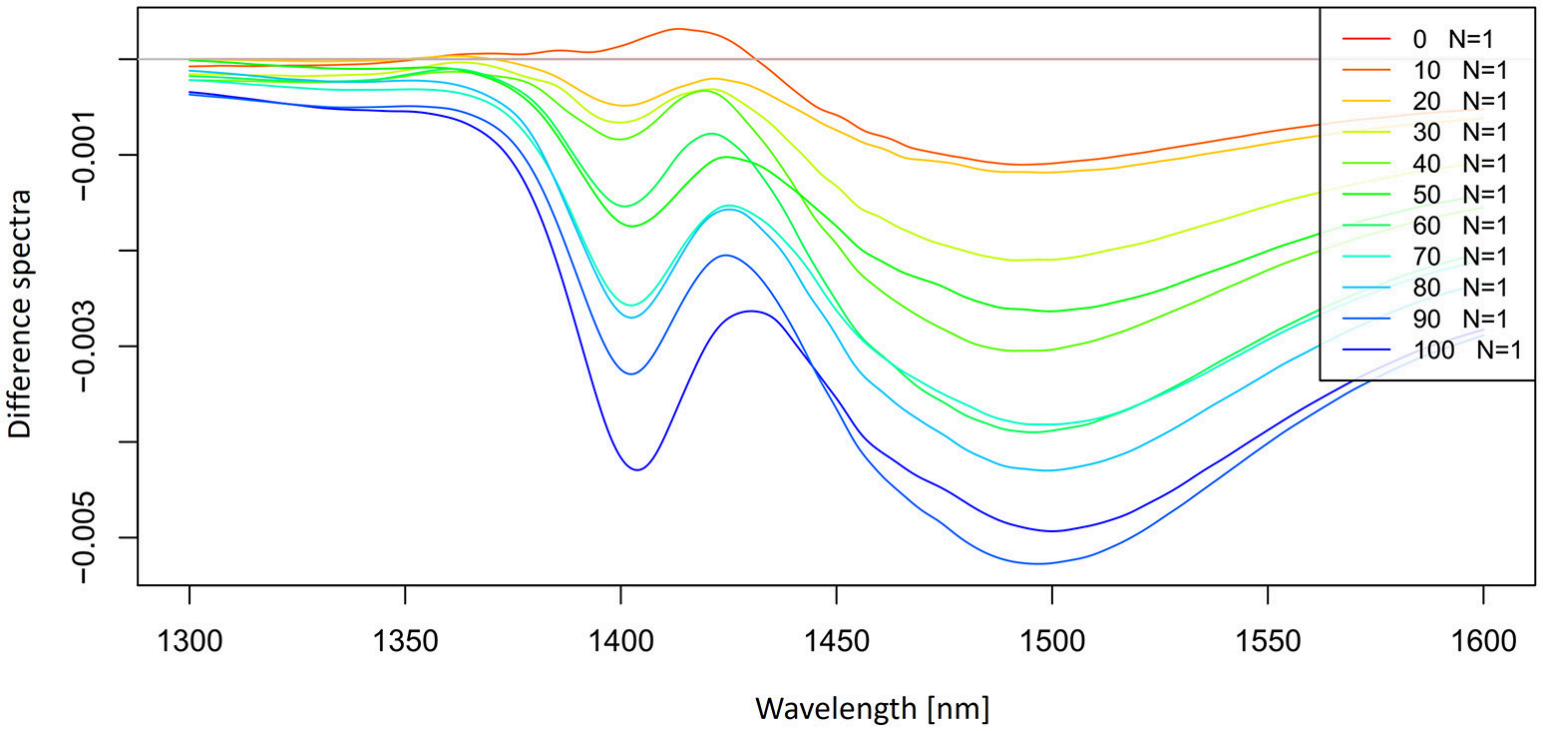
Smoothed (calculated with a Savitzky-Golay filter using 21 points) average difference absorbance (logT-1) spectra in the spectral range of 1,300–1,600 nm (OH first overtone) of Milli-Q water and aqueous solutions of potassium-chloride in the concentration range of 10–100 mM. Average spectrum of Milli-Q water was subtracted from the spectra of potassium-chloride solutions.

Let us now look at the examples of application of these preprocessing steps on the spectra of potassium chloride solutions. The smoothed spectra were calculated by using a Savitzky-Golay filter (2nd order polynomial fit and 21 points) and presented in Figure 3. Only the area of the first overtone 1,300–1,600 nm is plotted to provide a better visualization of how smooth the spectra should look. Next, a subtraction of the average spectrum of Milli-Q water from all the averaged spectra of potassium-chloride solutions was done and is presented in Figure 4.

The subtracted spectra reveled the existence of at least two major peaks under the broad overtone spectral curve of potassium-chloride solutions around 1,412 and 1,500 nm. It is also possible to observe a slight peak shift at 1,412 nm with increasing salt concentration.

**Figure 5:**
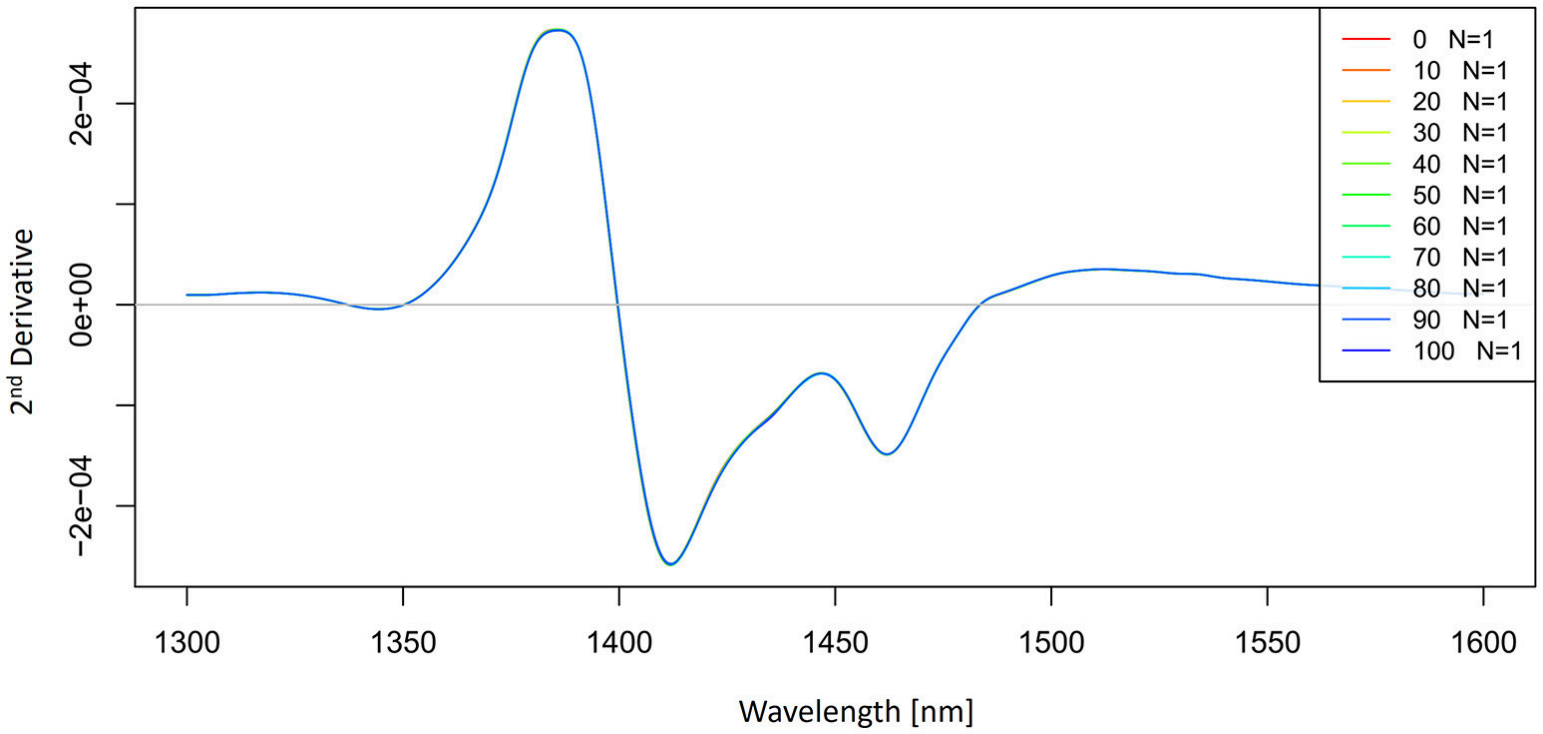
2nd derivative (calculated with a Savitzky-Golay filter using 2nd order polynomial and 21 points) average absorbance (logT-1) spectra in the spectral range of 1,300–1,600 nm (OH first overtone) of Milli-Q water and aqueous solutions of potassium-chloride in the concentration range of 10–100 mM.

The 2nd derivative spectra of potassium-chloride solutions were calculated by using a Savitzky-Golay filter (2nd order polynomial and 21 points) and presented in Figure 5. The second derivative spectra also indicate an existence of the band at 1,412 nm and we can also see the second band located at 1,462 nm.

With these simple preprocessing steps, we have so far identified at least two water absorbance bands activated by salt perturbation.

## Chemometrics- the importance of consistency

Similar to the classical spectroscopy, the use of chemometrics methods is a crucial part of the aquaphotomics data analysis as well. It includes many well-known exploratory, classification and regression methods depending on the objective of the experiment.

Principal components analysis (PCA) (Cowe and McNicol, 1985) is one of the most useful and probably mostly commonly used exploratory technique in spectroscopy during the early stages of data analysis. Its objective is to determine a possible relationship between samples, i.e., to provide the first clues about major directions and sources of variation in the dataset. It compresses data by constructing new variables and the results are presented in scores and loadings plots. The scores plots visualize the spectra in the form of scores in the transformed space of newly constructed variables—principal components, while the corresponding loadings plots denote the contributions of original variables—wavelengths. The novelty of PCA application in aquaphotomics analysis is that a particular attention is given to the analysis of all loading vectors as they can reveal activated water absorbance bands.

**Figure 6:**
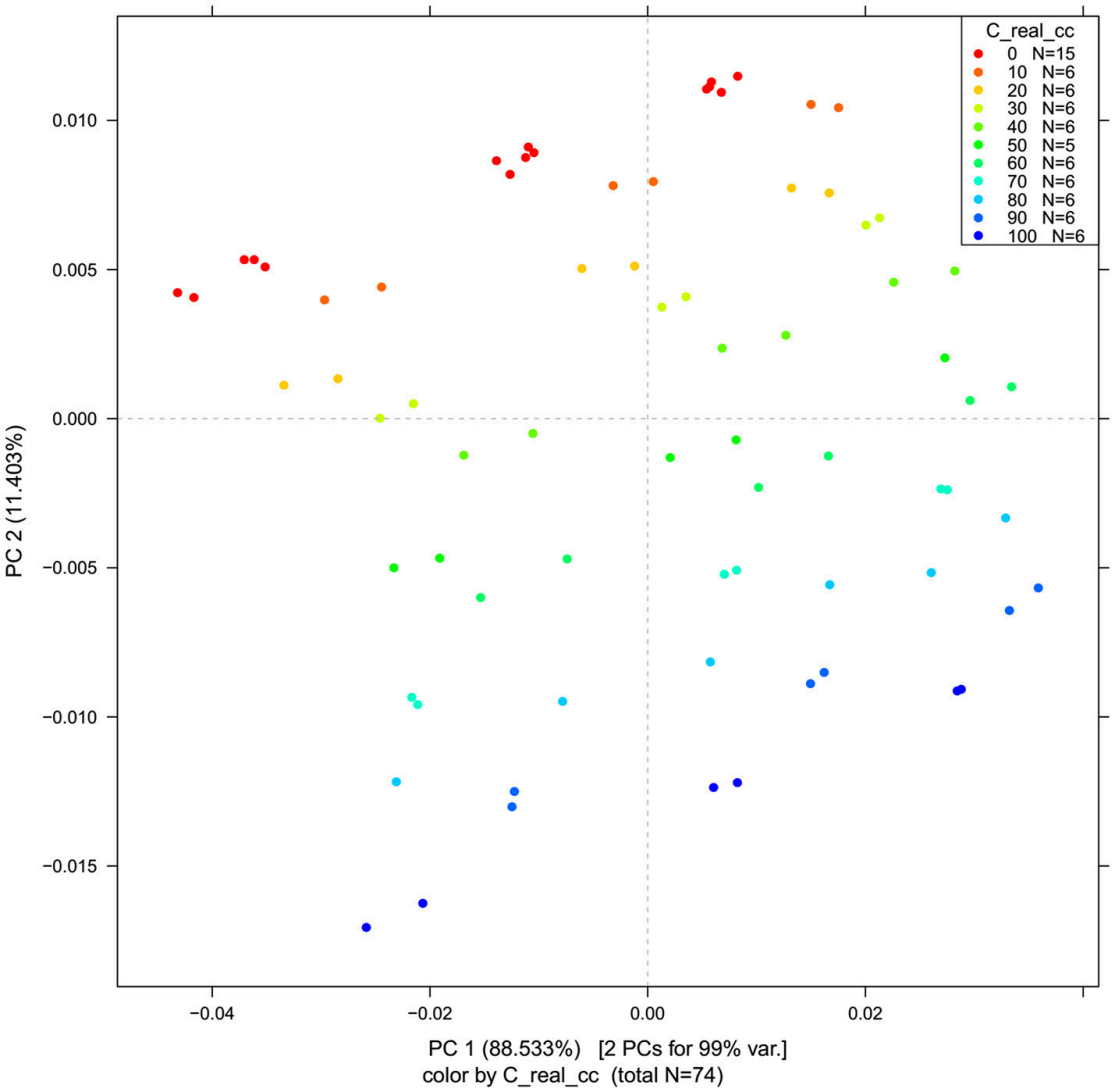
PCA analysis of Milli-Q water and aqueous solutions of potassium-chloride in the concentration range of 10–100 mM derived from the smoothed (calculated with a Savitzky-Golay filter using 2nd order polynomial and 21 points) and MSC transformed absorbance (logT-1) spectra in the spectral range of 1,300–1,600 nm (OH first overtone)—Scores plots for the first two principal components.

**Figure 7:**
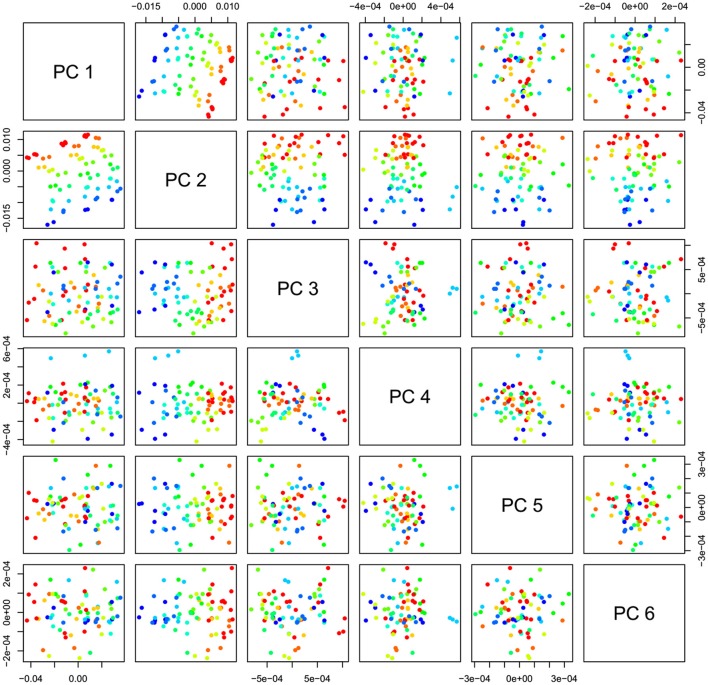
PCA analysis of Milli-Q water and aqueous solutions of potassium-chloride in the concentration range of 10–100 mM derived from the smoothed (calculated with a Savitzky-Golay filter using 2nd order polynomial and 21 points) and MSC transformed absorbance (logT-1) spectra in the spectral range of 1,300–1,600 nm (OH first overtone)—Scores plots for the first six principal components.

**Figure 8:**
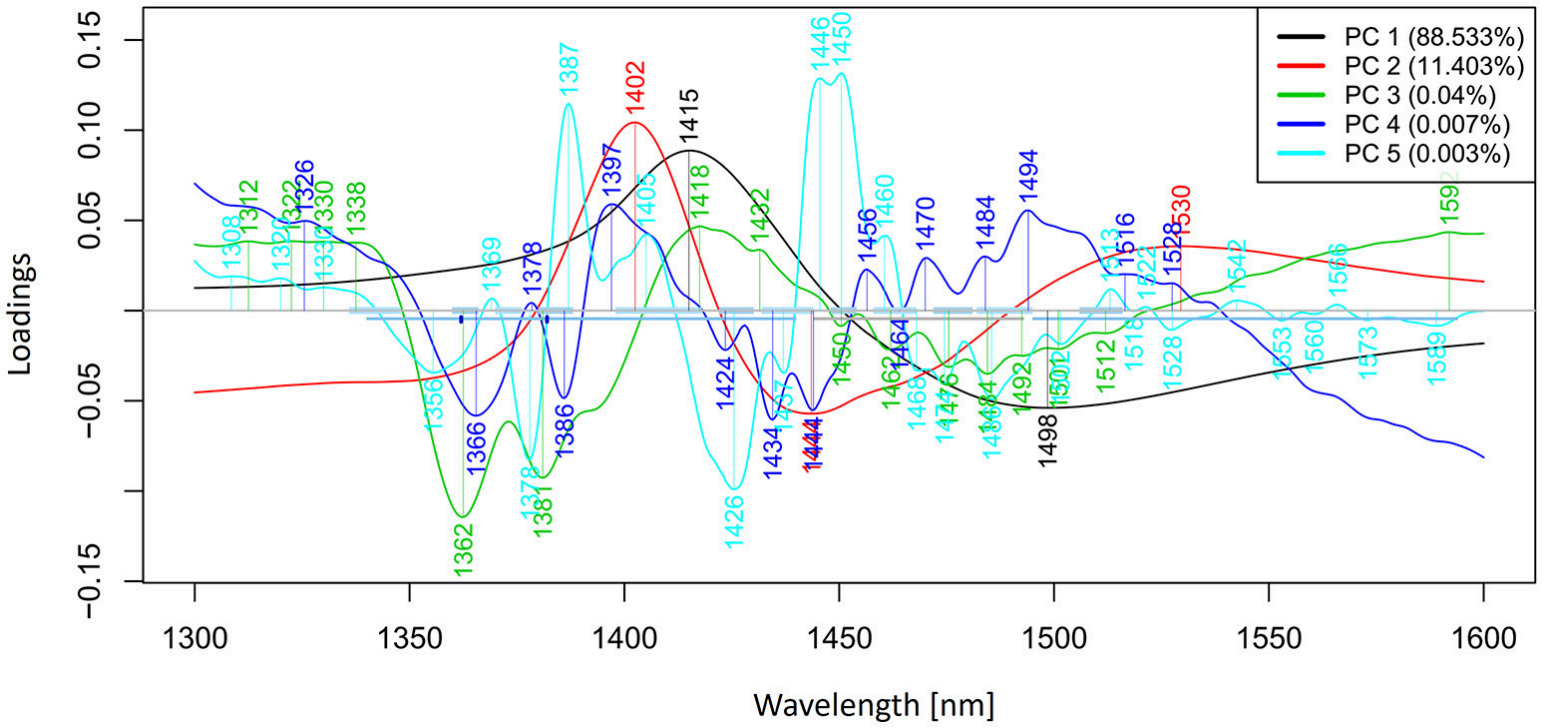
PCA analysis of Milli-Q water and aqueous solutions of potassium-chloride in the concentration range of 10–100 mM derived from the smoothed (calculated with a Savitzky-Golay filter using 2nd order polynomial and 21 points) and MSC transformed absorbance (logT-1) spectra in the spectral range of 1,300–1,600 nm (OH first overtone)—Loadings plot.

PCA in the case of our salt dataset was used to describe multidimensional patterns in the spectral data and discover outliers. PCA data presented in the scores (Figures 6, 7) and loadings plots (Figure 8) reveal major sources of variation in the data. The first two principal components describe more than 99.9% of variation in the dataset. The first principal component, whose loading shows two dominant features (a peak positive peak at 1,415 nm and a negative peak at 1,498 nm), is related to changes in water matrix caused by consecutive illumination. This effect is similar to that of temperature (Segtnan et al., 2001) in that free or weakly hydrogen bonded species absorbing at 1,415 nm increase at the expense of strongly hydrogen bonded water molecules absorbing at 1498 nm. The second principal component, which explains 11.403% of variation, shows the influence of concentration. It can be seen from the PC1-PC2 scores plot that while the scores move toward the negative part of the PC2 with increasing concentration, the pure water scores are entirely located in the positive part of this PC. The loading vector of PC2 presented in Figure 8 reveals major water absorbance bands affected by the presence of salt in water i.e., 1,402, 1,444, and 1,530 nm. Regarding loading vectors, it is very important to look at all PC loadings since changes in water are very subtle and might be also described by a higher number of PC loading vectors.

The next steps of the analysis depend on the objective of the experiment. They can involve classification methods to group samples together according to their spectra, or regression methods to link sample spectra to some quantifiable properties (Roggo et al., 2007). The application of these methods in aquaphotomics analysis does not differ much as compared to the classical NIR applications. However, the unique characteristics for the aquaphotomics approach are as follows.

First, the initial step of the aquaphotomics approach involves qualitative analysis. This step may include the application of PCA or some unsupervised classification analysis, performed with the objective of data exploration and better understanding of spectral variability. This step may even include some preliminary regression analysis, which can show very poor prediction results and non-linearity existence. However, it can provide information about the existence of natural clusters of samples indicating the need for separate modeling for different groups of samples thus discovered. For example, the most accurate prediction of milk components such as protein, lactose and fat in cow milk was achieved when the models were separately built by using milk spectra from healthy and mastitis animals (Tsenkova et al., 2001a,c). A subtraction of the averaged spectra of these two groups will give us the first information about the “important” WAMACS to be used in further analysis. The presence of mastitis disease (bacterial infection) significantly alters the structure of water in milk and milk composition, causing non-linearity in the regression models if the spectra of healthy and mastitis animals are used together. In this case, separately built regression models form a part of the aquaphotome database, where a different regression model is applicable depending on the physiological status of the animal. In this respect, aquaphotomics does not aim nor considers it possible to build global models. This is especially true in the analysis of biological systems that are far too complex to be described with only one model.

Second, the most important feature of aquaphotomics analysis is the special attention paid to original and transformed spectral vectors as well as model outputs. This reveals the contribution of original variables—wavelengths, to model development and tracks consistently repeating variables. The identified variables with high contribution, which constantly repeat through all the steps of aquaphotomics analysis, are the most informative ones. For aquaphotomics, these variables are the places in the spectra, where various water molecular conformations absorb. Their identification is crucial for better understanding of the aqueous system and response of its water matrix to the perturbation. In other words, the variables, which consistently appear in all aquaphotomics analysis (i.e., in subtracted spectra or transformed spectra, spectral derivatives, model outputs in the form of PCA loadings, PLSR regression vectors, SIMCA discriminating powers etc.), are the locations of water absorbance bands, where spectral variations under controlled and uncontrolled perturbations could be observed. If they persistently and consistently appear through all of the analysis, we can consider these water absorbance bands as activated.

**Figure 9:**
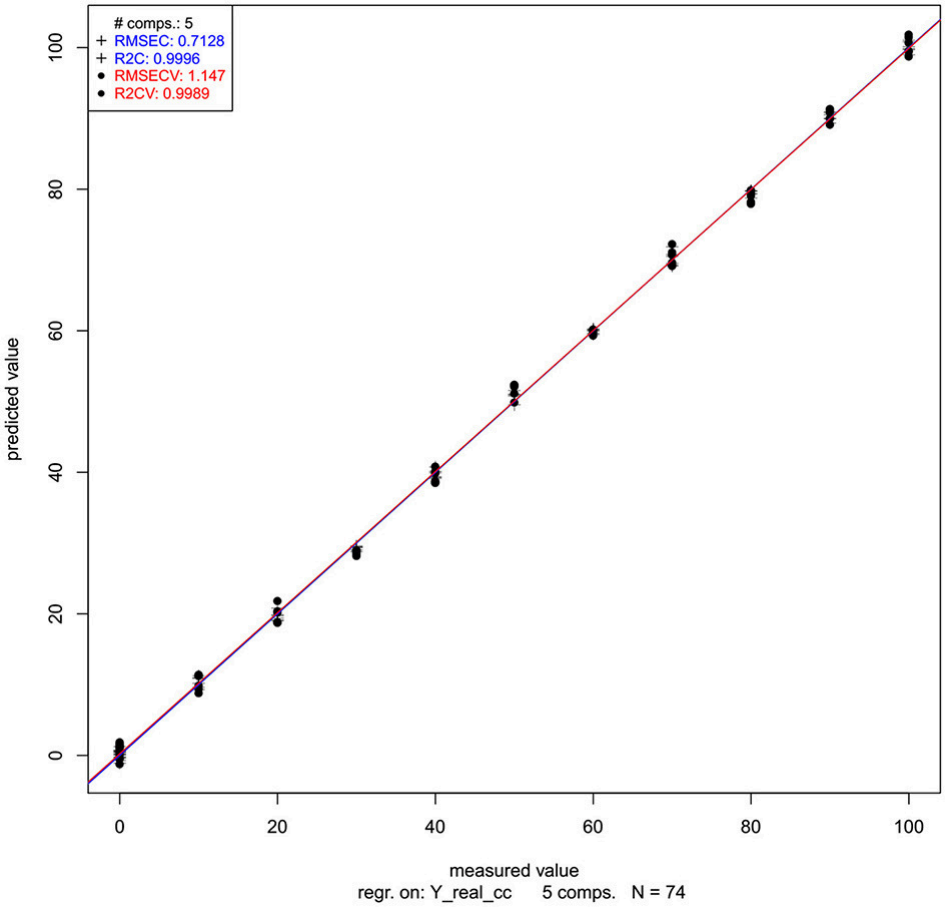
PLSR analysis of Milli-Q water and aqueous solutions of potassium-chloride in the concentration range of 10–100 mM derived from the smoothed (calculated with a Savitzky-Golay filter using 2nd order polynomial and 21 points) and MSC transformed absorbance (logT-1) spectra in the spectral range of 1,300–1,600 nm (OH first overtone) built for the prediction of potassium-chloride concentration: Y fit of training and one-sample-out cross-validation.

**Figure 10:**
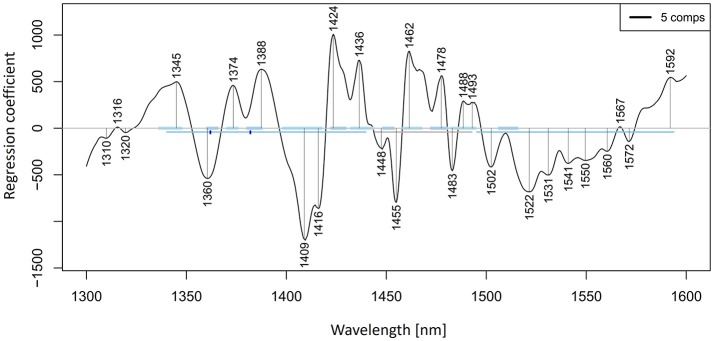
PLSR analysis of Milli-Q water and aqueous solutions of potassium-chloride in the concentration range of 10–100 mM derived from the smoothed (calculated with a Savitzky-Golay filter using 2nd order polynomial and 21 points) and MSC transformed absorbance (logT-1) spectra in the spectral range of 1,300–1,600 nm (OH first overtone) built for the prediction of potassium-chloride concentration: Regression vector.

Let us now look at the PLSR application on our salt dataset. The regression was performed on previously smoothed (Savitzky-Golay filter, 2nd order polynomial, 21 points) and MSC transformed spectra in the spectral range of 1,300–1,600 nm to build a model for prediction of potassium-chloride concentration. The results of PLSR analysis are presented in Figures 9, 10, showing a close correlation and a relatively low error of cross-validation using five latent variables (*r*^2^ = 0.9989, RMSECV = 1.147 mM, Figure 9). The main absorbance bands showing a significant weight in the PLS regression vector (Figure 10) match very well with those found in the previously applied methods, and all belong to the ranges of WAMACS found in the first overtone of water (Tsenkova, 2009). The favorable prediction results are not surprising since it is well established that salts influence the spectrum of water and these changes can be used for prediction of salt concentration (Grant et al., 1989; Gowen et al., 2015). Because salts do not absorb the NIR light, these results and the previously mentioned studies demonstrate the feasibility of aquaphotomics water-mirror approach. In other words, the absorbance bands of water can be used to obtain indirectly the information about changes in solute concentrations.

It is worth mentioning that the analysis may include several more chemometrics methods that can also contribute to the identification of water absorbance bands activated by the perturbation of interest.

Employing discriminant analysis such as Partial Least Squares Discriminant Analysis (PLS-DA) (Martens and Martens, 2001) for discriminating between solvent and solutions can help in gaining more insight about how the solutes affect the water matrix of the solvent. For example, this method was employed to discriminate between solvent and pesticide–containing solutions (Gowen et al., 2011). Examination of the regression vectors of PLS discriminant analysis provides an additional help in revealing water absorbance bands activated by the presence of solutes.

Similarly, Soft Modeling of Class Analogies (SIMCA) (Wold and Sjöström, 1977) can be employed for the same purpose. The discriminating power of SIMCA analysis, in that case, reveals water absorbance bands with the highest discriminating power which distinguishes between pure solvent and solutions. One such example can be found in an aquaphotomics study concerned with measurements of different saccharides at millimolar concentrations (Bázár et al., 2015). Sometimes, both discrimination methods (SIMCA and PLS-DA) are employed for the same purpose of discriminating the solvent from the solutions and the discovery of additional information about activated water absorbance bands by solutes. In a study concerned with the detection of UVC damaged DNA, both PLS-DA and SIMCA were applied to distinguish between non-irradiated and UVC-irradiated DNA solutions (Goto et al., 2015). Applying two chemometrics methods for the examination of one aspect of the experimental study demonstrates the stability of the applied methodology, namely, consistency in results.

Both the SIMCA and PLS-DA methods are naturally used in most cases when the objective of the study is discrimination between different samples. For classification and discrimination purposes in aquaphotomics, the most commonly used methods are SIMCA and PLS-DA. The SIMCA method was employed, e.g., for discrimination between healthy and mosaic virus infected soybean plants (Jinendra et al., 2010), for discrimination between healthy and mastitic animals based on the spectra of urine, blood and milk of dairy cows (Tsenkova, 2004), for discrimination between different brands of commercially available mineral waters (Munćan et al., 2014), for discrimination of different bacteria strains (Remagni et al., 2013; Slavchev et al., 2015, 2017) and others. The PLS-based discriminant analysis was applied for discrimination between irradiated and non-irradiated DNA solutions (Goto et al., 2015), discrimination between solvents and pesticides containing solutions (Gowen et al., 2011), and discrimination between worn and new soft contact lenses based on conventional hydrogels (Šakota Rosić et al., 2016).

Quantitative aquaphotomics analysis usually includes partial least squares regression (PLSR) (Martens and Martens, 2001) or principal component regression (PCR) (Næs et al., 2002). The principal uniqueness of the aquaphotomics approach in the application of these two methods is the utilization of water absorbance bands for indirect quantification of analytes in water, which change the water matrix. The feasibility of this approach was demonstrated in a study whose objective was quantification of different types of salt in water solutions (NaCl, KCl, MgCl_2_, and AlCl_3_), where the overall detection limit of 1,000 ppm was reported (Gowen et al., 2015). The experiment was reproduced in three independent laboratories by using 3 different spectrometer systems and in different ambient conditions. The reported detection limit of 1,000 ppm indicates that under specified conditions, the aquaphotomics approach substantially improved the detection limit for NIRS (around 5 times) (Pasquini, 2018).

Using an aquaphotomics approach, PLSR gave excellent results for quantification of various analytes in water solutions such as sugars [glucose, fructose, sucrose and lactose and their mixtures (total sugar and each sugar concentrations)] (Bázár et al., 2015), insulin protein (Chatani et al., 2014), DNA, isolated cyclobutane pyrimidine dimers, and UVC-irradiation dose (Goto et al., 2015). The same approach also provided a favorable accuracy of measurements in more complex biological samples, such as human serum albumin (HSA) and γ-globulin in phosphate buffer solutions (Murayama et al., 1998), urinary estrone-3-glucuronide (E_1_G) concentrations in urine of giant pandas (Kinoshita et al., 2010, 2012), HIV virus in human plasma (Sakudo et al., 2005), somatic cell counts in cow milk samples (Tsenkova et al., 2001a; Tsenkova, 2004), as well as fat, lactose, protein and urea nitrogen content of milk (Tsenkova, 2004).

Very recently, a critical review on NIRS and its modern perspectives expressed concerns regarding the capability of aquaphotomics for measurement of analytes in very low concentrations, given the fact that the concentrations of 5,000 ppm (mg L^−1^) or 0.5% (w/v) are roughly regarded as a common limit of quantification for NIRS (Pasquini, 2018). Capability comparison of the traditional NIRS and aquaphotomics approach is based on an incorrectly assumed equivalence. While the established limit of detection for the traditional approach is based on the utilization of absorbance bands of analytes in the NIR region, the aquaphotomics approach utilizes water absorbance bands. In this sense, the quantification of analytes is based on entirely different principles, and as such, logically offers different limits of detection. Different approaches and their accuracy of detection were well demonstrated in studies on the measurement of concentrations of polystyrene particles in water (Tsenkova et al., 2007b). When the first overtone of water (i.e., aquaphotomics approach) was used to develop a model for low concentrations of polystyrene particles in aqueous suspensions (1 – 0.0001%), the measurements achieved a high accuracy even in the case of very low concentrations. However, when the traditional approach was applied and measurements were based on the polystyrene band near 1,680 nm (C-H stretching from aromatic C-H (2ν) (Workman, 2016)—i.e., decreasing particle concentration led to a substantial decrease in accuracy of prediction.

Aquaphotomics can work with very water-rich systems. The intensity of water bands in the NIR spectra of such systems is much stronger than that of any constituent (Tsenkova, 2004), especially if they are in very low concentrations. The possibility of detecting and measuring such low concentrations arises from the fact that every molecule of analyte is hydrated with an abundance of water molecules, which adapt to its structure and assume various conformations that can be observed based on their respective absorbance bands in the NIR region. Since many water molecules are involved with hydration of just one molecule of analyte, the water acts as a sort of amplifier, and instead of measuring analytes directly, the information on their concentration is obtained indirectly by measuring changes in always abundant solvent molecules.

NIR spectroscopy as a non-destructive tool offers the advantage of *in vivo* spectral monitoring of living objects. Aquaphotomics combined with time-resolved NIR spectroscopy allows a better understanding of biological functions and underlying water dynamics.

One of the excellent methods for exploring water dynamics is generalized two-dimensional (2D) correlation spectroscopy (Noda et al., 1995; Liu et al., 1996). In 2D correlation spectroscopy, an external perturbation is applied to a system during spectral measurements, which enables exploration of spectral signals as a function of time or perturbation level (where perturbation can be a number of consecutives, temperature, concentration etc.). This method has significant advantages over one-dimensional spectra. Spreading the spectral region over another dimension allows a deconvolution of overlapped bands and monitoring a specific order of spectral intensity changes. Moreover, 2D correlation spectroscopy offers the possibility of investigating various intra- and inter-molecular interactions through selective correlation of peaks. This technique, in addition to PCA, considerably contributed to the understanding of the structure of liquid water (Segtnan et al., 2001). Furthermore, it was applied for extraction of useful information from NIR spectra of protein aqueous solutions during heat-induced denaturation of ovalbumin (Wang et al., 1998) and acid-induced denaturation of human serum albumin (Murayama et al., 2000). The method can be applied even in the case of complex biological fluids such as milk (Czarnik-Matusewicz et al., 1999; Tsenkova, 2004) or complex biological samples such as fruits (Giangiacomo et al., 2009). 2D correlation analysis was also employed for the investigation of wafer etchant solutions composed of several inorganic acids (HCl, H_2_SO_4_, H_3_PO_4_, and HNO_3_) (Chang et al., 2018). This study, using a typical water-mirror approach, applied 2D correlation analysis to examine NIR water bands perturbed by four acids and determined their dissimilar characteristics. The results showed that components with higher acidity in single-component samples perturbed water hydrogen bond network more significantly, and in turn allowed more accurate concentration measurements. Heterospectral correlation (Noda and Ozaki, 2004) i.e., investigation of correlation between water absorbance bands in different regions of the electromagnetic spectrum (IR and NIR) or by different techniques (NIR and Raman spectroscopy) can significantly contribute to the development of aquaphotomics through discovery and identification of new water absorbance bands. However, it should be pointed out that there is one inherent weakness of the method, i.e., high level of sensitivity to noise.

Other approaches for examination of water dynamics are also often in use. For example, plotting SIMCA interclass distance as a function of time revealed time-dependent spectral dynamics of virus infection in soybean plants (Jinendra et al., 2010). The SIMCA interclass distance between the groups of infected and non-infected plants showed small values of around 1.2 (2 weeks after inoculation), then gradually decreased to the lowest value of 0.8 (3 weeks after inoculation). After this critical point, the value of interclass distance increased steadily. Thus, revealed water dynamics mirrored the dynamics of viral infection where, due to the defense reaction from the plants, the disease impact was initially suppressed exactly 3 weeks after inoculation. The same approach was utilized in a study of the ovulation period in giant pandas (Kinoshita et al., 2010). Interclass distances were calculated between spectra of urine collected each day in the time series and urine spectra collected at the first day of investigation when the female animals had been in an estrous state. This analysis showed that the SIMCA distance between these two groups increased simultaneously with an increase in E1G concentration, a major estrogen metabolite excreted in the urine during estrus. Another study was concerned with investigation of protein fibrillation and employed spectral monitoring of water structural changes in real time during fibrillation of insulin (Chatani et al., 2014). This study monitored the process of fibrillation of insulin indirectly by monitoring water molecular structure dynamics in the region of the first overtone (1,300–1,600 nm), while the verification of formation of fibrils was performed by two methods i.e., FTIR spectroscopy and Atomic Force Microscopy. The PCA analysis of NIR spectra of protein solutions found that for the first two PCs, score changes can be mainly attributed to a change in light scattering; however, the scores of PC3, when expressed as a function of time (in minutes), showed a time course of changes in water structure coinciding well with the proposed nucleation, elongation and equilibrium phase of protein fibrillation (Chatani et al., 2014). It is worth mentioning that other ways of exploring water dynamics are possible. For example, expressing SIMCA interclass distance as a function of consecutive illumination or temperature can reveal different responses to perturbation in different samples, which otherwise, without perturbation, may be difficult to discriminate due to a high similarity. Also, expressing SIMCA interclass distance between solvent and solutions of varying concentrations, as a function of concentration, may reveal concentration ranges in which solutes have structure-breaking and structure-making effect, thus indicating the need for building separate regression models for different ranges of concentrations.

Recently, several novel chemometrics methods were introduced to aquaphotomics studies. Multivariate curve resolution-alternating least squares (MCR-ALS) was applied to characterize the effects of temperature and salt perturbations on the NIR spectra of water in order to gain more insight into hydrogen bonding (Gowen et al., 2013). This advanced data analysis technique applies a factor model approach with the objective of recovering pure concentration and spectral profiles of the components in complex mixture systems without any prior knowledge of these features (Czarnecki et al., 2015). To perform MCR, however, one has to estimate firstly a number of significant components, usually based on PCA analysis, In contrast to PCA, MCR can provide results that have actual physical and chemical meaning (Czarnecki et al., 2015). The “components” in terms of water structures could be interpreted as the changing forms of water when perturbations were applied. Three distinct components were found with varying temperature dependence in the range 30-45°C in the region of first overtone of water, while different salts and salt concentration levels affected the water hydrogen bonded network in different ways according to its acidity (Gowen et al., 2013). By resolving different systems into idealized pure components, MCR-ALS allowed better examination of water molecular matrix and resulted in the conclusion that the water structure can be reasonably interpreted as a multi-state system.

Evolving factor analysis (EFA) was applied for exploration of hydration and secondary structures of bovine serum albumin in aqueous solutions (Yuan et al., 2003). Application of this method allowed an extraction of spectral information, which indicated significant changes of bovine serum albumin in secondary structure. The application of independent component analysis (ICA) was reported in spectroscopic analysis of hydrogen bonding in water-acetone mixtures for resolving the spectra to independent components and obtaining their concentration profiles (Monakhova et al., 2014). A Gaussian fitting method was applied to study glucose-induced variation of water under temperature perturbation (Cui et al., 2016). This method, applied on a NIR difference absorbance spectra (in region 700–1,100 nm), helped identify and quantify 16 inorganic salts in water in the concentration range from 30 to 500 mM (Steen et al., 2015).

A series of articles were also published on employing and developing various chemometrics methods specifically for temperature-perturbed samples (Peinado et al., 2006; Shao et al., 2010, 2018; Kang et al., 2011; Shan et al., 2015; Cui et al., 2017b). Instead of trying to eliminate the influence of temperature, a Parallel Factor (PARAFAC) model was used to extract and separate relevant sources of both physical and chemical information (Peinado et al., 2006). PARAFAC analysis was also used to rationalize concentration-dependent peak shifts and quantification of different water species in acetone (Andrews et al., 2014), and also for a quantitative analysis of the NIR spectra of temperature-perturbed mixtures, water-ethanol-propanol and water-ethanol-glycerin (Peinado et al., 2006). Multilevel simultaneous component analysis (MSCA) has been applied to the investigation of a relationship between temperature and NIR spectra of different samples in different concentrations: water-ethanol-isopropanol, (Shan et al., 2015) and water-glucose (Cui et al., 2017a) under temperature-perturbation. This method was proposed specifically for analyzing multivariate data at different levels (Timmerman, 2006). The method offers a unique way to study the composition of solvent, temperature effect and quantitative analysis (Shan et al., 2015). Cui et al. tested three high-order chemometric algorithms: multiway principal component analysis (MPCA) (Wold et al., 1987), parallel factor analysis (PARAFAC) (Bro, 1997) and alternating trilinear decomposition (ATLD) (Wu et al., 1998) in the analysis of temperature-dependent NIR spectra of binary and ternary water-alcohol mixtures (Cui et al., 2017b). All three algorithms proved to be very powerful tools for capturing temperature– and concentration–induced spectral variations, from which a structural variation could be observed and a quantitative determination performed. Another work of Shao et al. proposes mutual factor analysis (MFA) for quantification based on temperature-dependent NIR spectra (Shao et al., 2018). In this work, multi-component mixtures were analyzed for quantification of components and better understanding of molecular interactions in solutions. From the spectra of water–glucose mixtures, both spectral variations induced by temperature and concentration were obtained while serum samples were used for method validation (Shao et al., 2018).

The ultimate choice of chemometrics method to be applied in aquaphotomics analysis depends on the type of the aqueous system explored, spectral dataset and the research objective. Obviously, there are many chemometric methods available. The important aspect of every aquaphotomics analysis is emphasis on consistency so that each preprocessing method, conventional spectroscopic method or chemometrics method applied to extract the information from water spectra can contribute to the development of an emerging aquaphotome. Each step of aquaphotomics data analysis is important, because it can contribute to better understanding of the complexity of aqueous systems, irrespective of chemometrics method applied.

With reference to our example of potassium chloride solutions, after examining the raw spectra, difference spectra, second derivative spectra, loadings of PCA analysis and regression vector of PLSR analysis, we have identified the main water absorbance bands activated by the perturbation of potassium chloride in the concentrations up to 100 mM. The last step of analysis for our worked-out example is to represent water absorbance spectral patterns using aquagrams.

## Water spectral pattern represented by aquagrams

### Classic aquagrams

In data analysis, many situations arise where data visualization is helpful, even essential, for better understanding. In aquaphotomics, the need arose for a clear and comprehensive graphical representation of the water spectral patterns as well as for their easy comparison. That is why the aquagrams were introduced (Tsenkova, 2010).

When activated water absorbance bands are found based on the previously described steps, the last step is to apply MSC or SNV transformation of the raw spectra, and extract the absorbance at selected activated water bands. Thus, the calculated absorbance is normalized and averaged for different samples or sample groups, and the values are displayed on radial axes defined by the activated water absorbance bands in a radar chart.

The normalized absorbance is calculated as follows:

(1) $$\begin{array}{r} A_{\lambda }^{,}=\frac{A_{\lambda }-\mu_{\lambda }}{\sigma_{\lambda }} \end{array}$$

Where $A_{\lambda }^{,}$ - is a normalized absorbance displayed on the aquagram, *A*_λ_- absorbance after multiplicative scatter correction (MSC) or standard normal variate transformation (SNV), μ_λ_ – mean of all spectra for the examined group of samples after transformation, σ_λ_ – standard deviation of all spectra for the examined group of samples after transformation, λ – selected wavelengths chosen for display from activated water absorbance bands.

An exact number of axes as well as water absorbance bands will be chosen for display, depending on a specific system and perturbation; however, the axes always display various conformations of water molecules, making aquagrams very convenient tools for a quick insight into the water structure of the system. For the first overtone of water, the axes of the aquagram are usually based on previously discovered 12 WAMACs. The aquagrams are visually very convenient to allow a fast and comprehensive comparison of different systems or conditions of the same system by comparison of their WASPs.

As it can be seen from Equation (1), the classic aquagram is a relative construction, depending on the samples included in calculation. Also, it is a matter of choice whether the display of absorbance calculated based on the above equation is done by using a circular chart (radar chart) or a linear one. The package aquap2 offers both options (Pollner and Kovacs, 2016).

The more advanced version of a classic aquagram is an aquagram with confidence intervals (Pollner and Kovacs, 2016). This aquagram adds one more function, the possibility to observe whether the differences among WASPs presented in the aquagrams are statistically significant. This type of aquagram, in addition to averaged WASPs for selected groups of samples, displays its confidence intervals with 95% upper and lower limits, as calculated by using the Bootstrap method for data validation and uncertainty estimation (Davison and Hinkley, 1997; Pollner and Kovacs, 2016). With this novel function, the aquagrams with confidence intervals are not only convenient for visualization, but also especially suitable for classification and discrimination.

**Figure 11:**
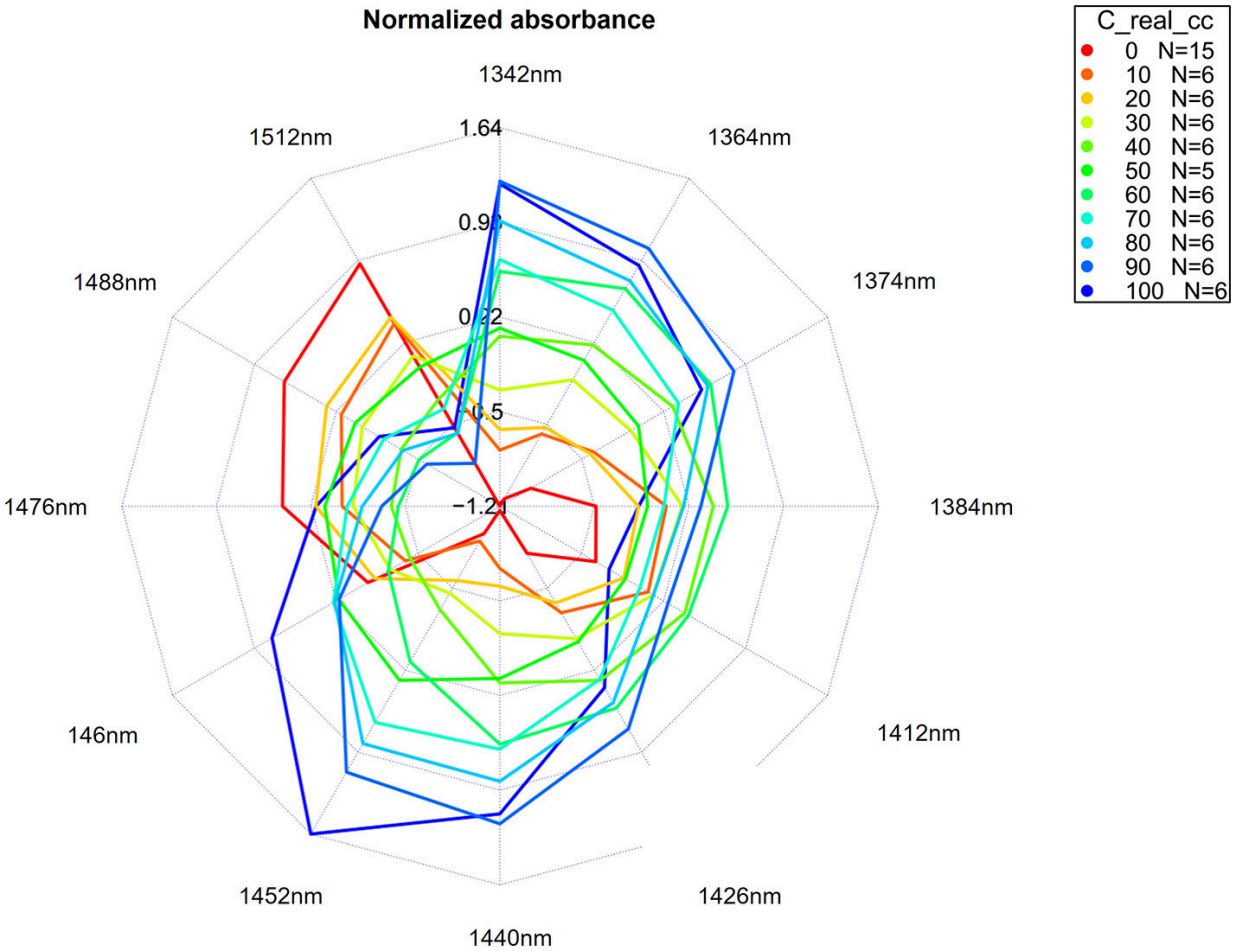
Aquagrams without confidence intervals of Milli-Q water and aqueous solutions of potassium-chloride in the concentration range of 10–100 mM calculated on the MSC transformed absorbance (logT-1) spectra in the spectral range of 1,300–1,600 nm (OH first overtone) using the “classic” mode.

**Figure 12:**
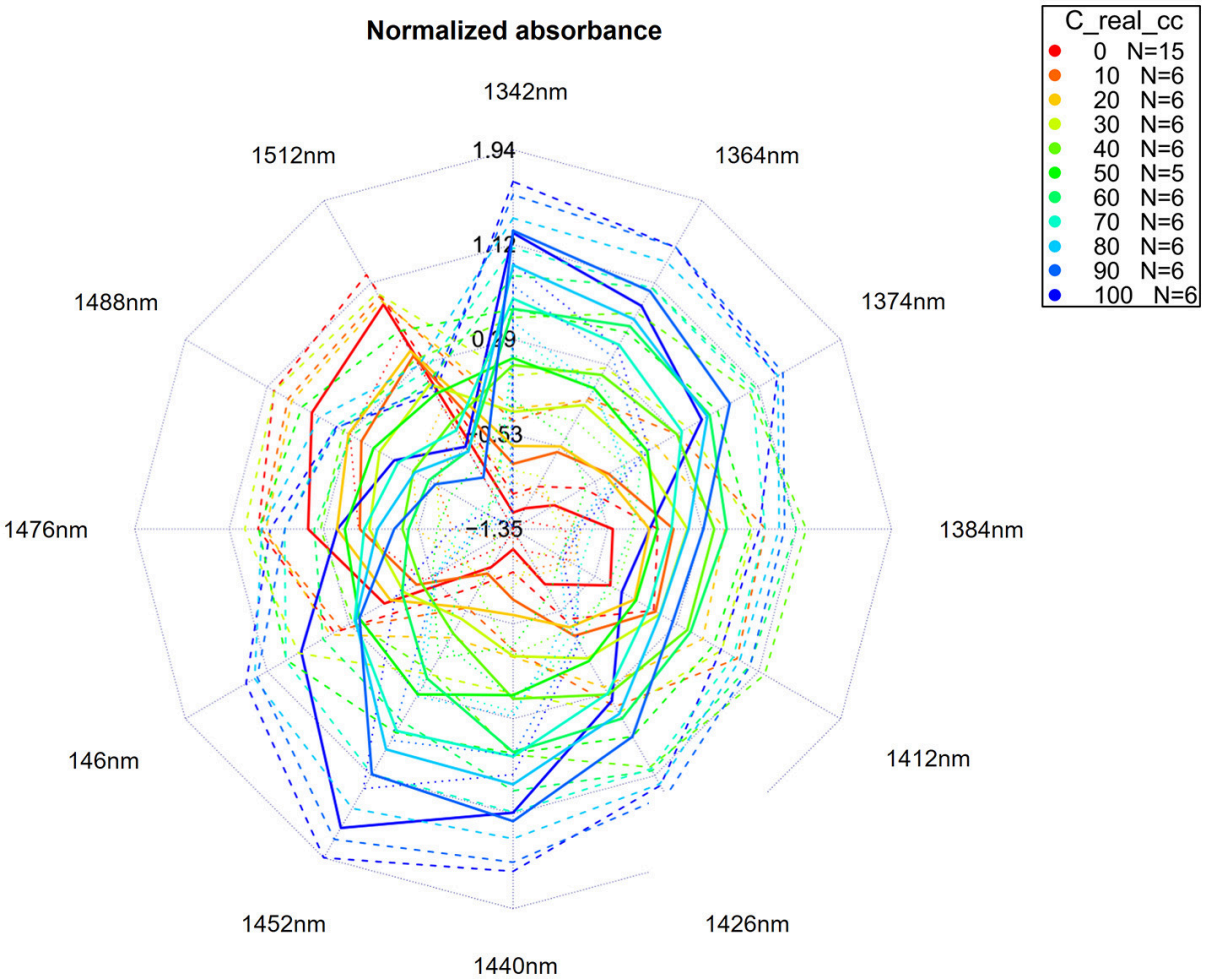
Aquagrams with 95% confidence intervals of Milli-Q water and aqueous solutions of potassium-chloride in the concentration range of 10–100 mM calculated on the MSC transformed absorbance (logT-1) spectra in the spectral range of 1,300–1,600 nm (OH first overtone) using the “classic” mode.

For our example dataset of potassium chloride solutions, after selecting wavelengths from the WAMACS regions in the 1st overtone of water based on the previous steps of the analysis, the classic aquagrams without and with confidence intervals, calculated by using aquap2 package, are presented in Figures 11, 12.

In both types of aquagrams, it is easy to observe a large difference between the spectral patterns of water (red line) and salt solutions. Increasing the concentration of salt in water leads to increased absorbance in the region between 1,342 and 1,374 nm which corresponds to C_1_, C_2_, and C_3_ WAMACS, i.e., absorbance of the free OH stretch (OH-(H2O)n, *n* = 1…4) (Xantheas, 1995; Robertson et al., 2003). An increase in the absorbance with increasing salt concentration can also be seen in the region stretching from 1,440 to 1,452 nm, i.e., C_7_-C_8_ WAMACS that are known as bands of water hydration (Gowen et al., 2009a) and water dimers (S_1_) (Segtnan et al., 2001; Cattaneo et al., 2009) and symmetric and asymmetric stretching of the first overtone of water (Siesler et al., 2008; Cattaneo et al., 2009; Gowen et al., 2009a). However, in the range between 1,476 and 1,512 nm, i.e., C_10_-C_12_, samples with higher salt concentration show lower absorbance values and this region is usually connected to strongly hydrogen bonded water (Segtnan et al., 2001; Tsenkova, 2009). The spectral pattern of salt solutions represented in the aquagrams shows that for the range of concentrations of salt under study, increasing salt concentration has a structure-breaking effect on water.

### Temperature-based aquagrams

In the previous section, we briefly mentioned that classic aquagrams are relative constructs, meaning that the WASPs displayed depend on the samples included in calculation. This is disadvantageous if the WASPs of samples or groups of samples ought to be compared over time or in different experiments. The development of a new temperature-based aquagram (Pollner and Kovacs, 2016) overcomes this difficulty by transformation of how spectral changes are expressed.

For the calculation of temperature-based aquagrams, it is necessary to first acquire a spectral library consisting of spectra of pure water (Milli-Q) at different temperatures covering a wider range of temperatures than the one expected to be used during the experiment. This created library, or so- called *reference dataset*, provides the basis for temperature aquagram calculation. The spectra from this dataset are to be compared with the spectra acquired during the experiment—*experimental dataset*, giving the ground to express the effect of certain perturbation on spectral pattern of experimental samples in terms of the effect of temperature on pure water spectra. In this way, the effect of any perturbation on samples can be expressed in the “temperature equivalent units,” in other words, changes in pure water spectra caused by temperature.

The calculation of a temperature-based aquagram is based on a comparison of areas covered by 12 WAMACS (Ci, *i* = 1, 12) coordinates in the region of the 1st overtone of water. The average spectra across all sample replicates and consecutive scans are calculated for the reference and experimental datasets. The area under the curve (AUC) for every single average spectrum for both reference and experimental datasets, at the wavelength range of each WAMACS (Ci) is calculated by taking into account the baseline estimated by linear fitting on the two edges of the first overtone region (i.e. through 1,300 and 1,600 nm points). The ratio of AUCs for every single water matrix coordinate and AUC for the first overtone region (i.e., 1,300–1,600 nm) are calculated for each averaged spectrum of both datasets in order to provide normalized values for comparison of reference and experimental datasets and to eliminate possible differences due to the scattering or path length differences. Using local polynomial regression for the reference dataset, a continuous array of values for the relative area of each Ci is calculated for a continuous temperature range chosen to include a specific temperature. In this way, a temperature calibration equation is obtained establishing a relationship between temperature and each Ci area, including the temperature at which the experiment was performed. When it is known how each Ci area for the pure water dataset is changed as a function of temperature, it is possible to pair these changes to spectral changes in the experimental dataset, i.e., to perform linking (mapping) and express the changes in Ci areas of the experimental datasets in the unit of temperature (degree Celsius) equivalent.

With this type of aquagram, it is also possible to include confidence interval limits. In that case, it is also necessary to perform transformation of upper and lower 95% confidence limits in the same manner just described above for the average spectra from the experimental dataset.

The whole calculation procedure for temperature-based aquagrams is implemented in the aquap2 package of R programing language (Pollner and Kovacs, 2016; R Core Team, 2017). An obvious disadvantage of temperature-based aquagrams is that they are based on previously discovered WAMACS regions in the first overtone of water (Tsenkova, 2010), meaning that at the moment this type of aquagram cannot be used for other windows of the electromagnetic spectrum where water absorbs.

**Figure 13:**
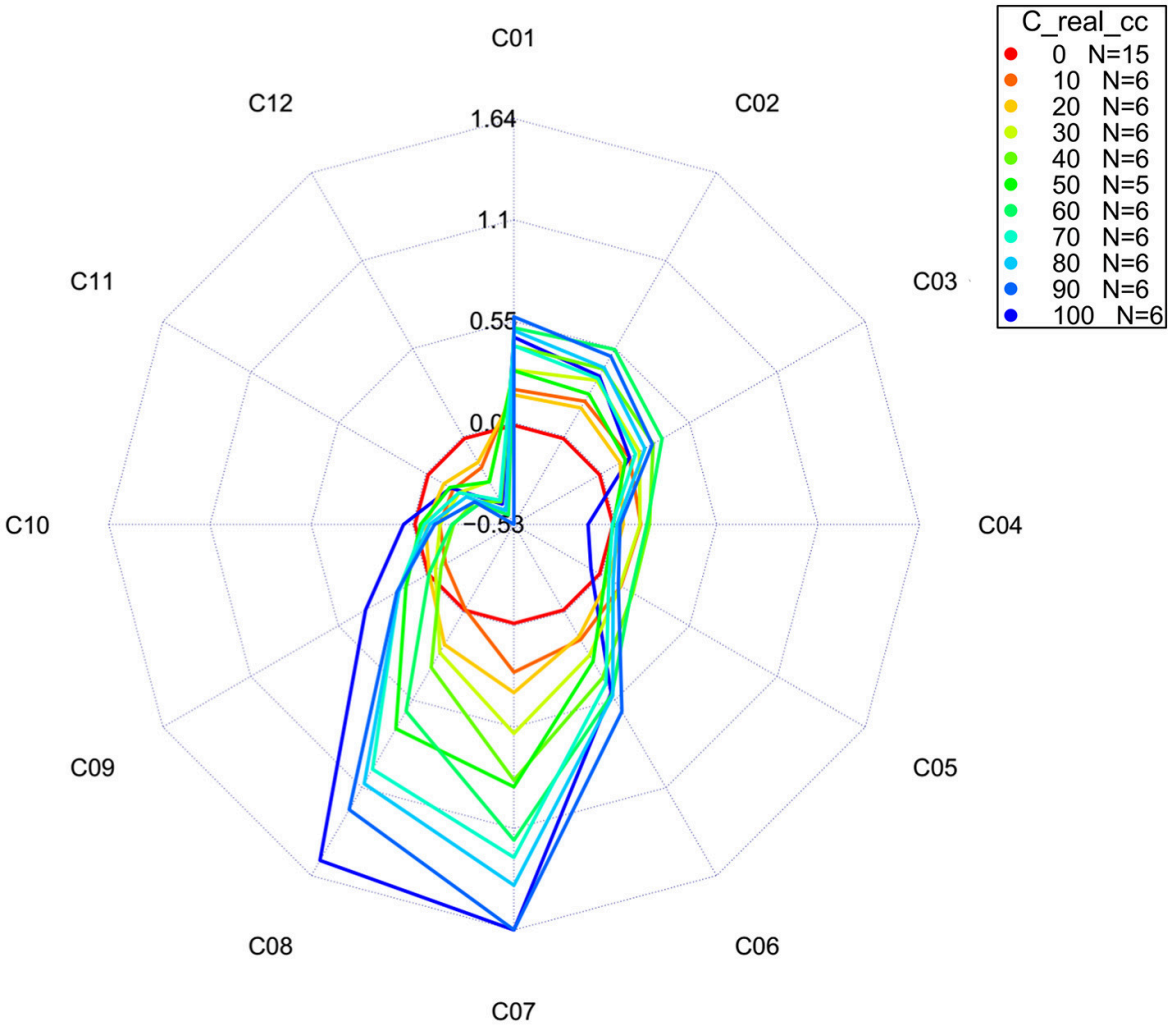
Aquagrams without confidence intervals of Milli-Q water and aqueous solutions of potassium-chloride in the concentration range of 10–100 mM calculated on the MSC transformed absorbance (logT-1) spectra in the spectral range of 1,300–1,600 nm (OH first overtone) using the “temperature-based” mode.

**Figure 14:**
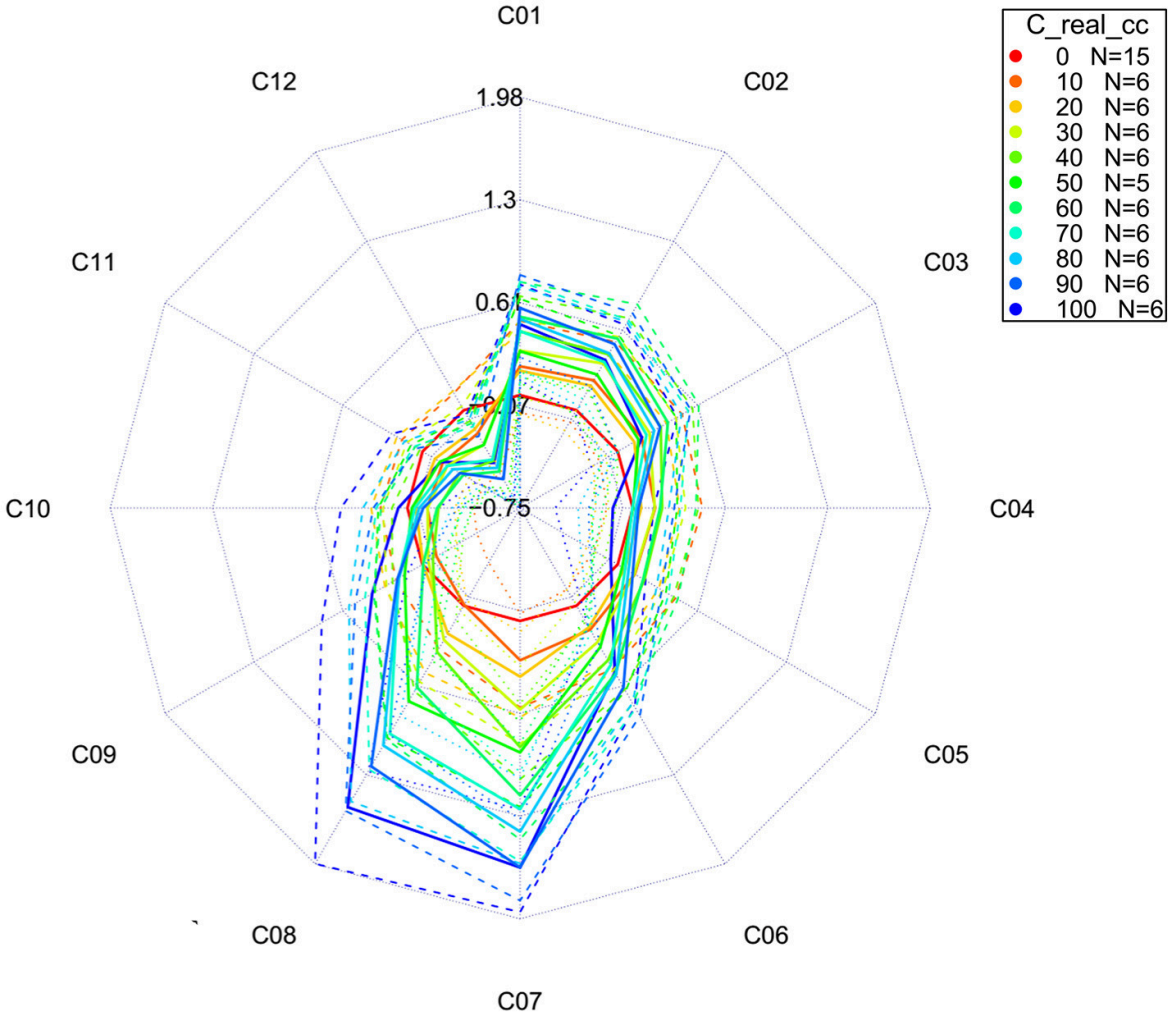
Aquagrams with 95% confidence intervals of Milli-Q water and aqueous solutions of potassium-chloride in the concentration range of 10–100 mM calculated on the MSC transformed absorbance (logT-1) spectra in the spectral range of 1,300–1,600 nm (OH first overtone) using the “temperature-based” mode.

**Figure 15:**
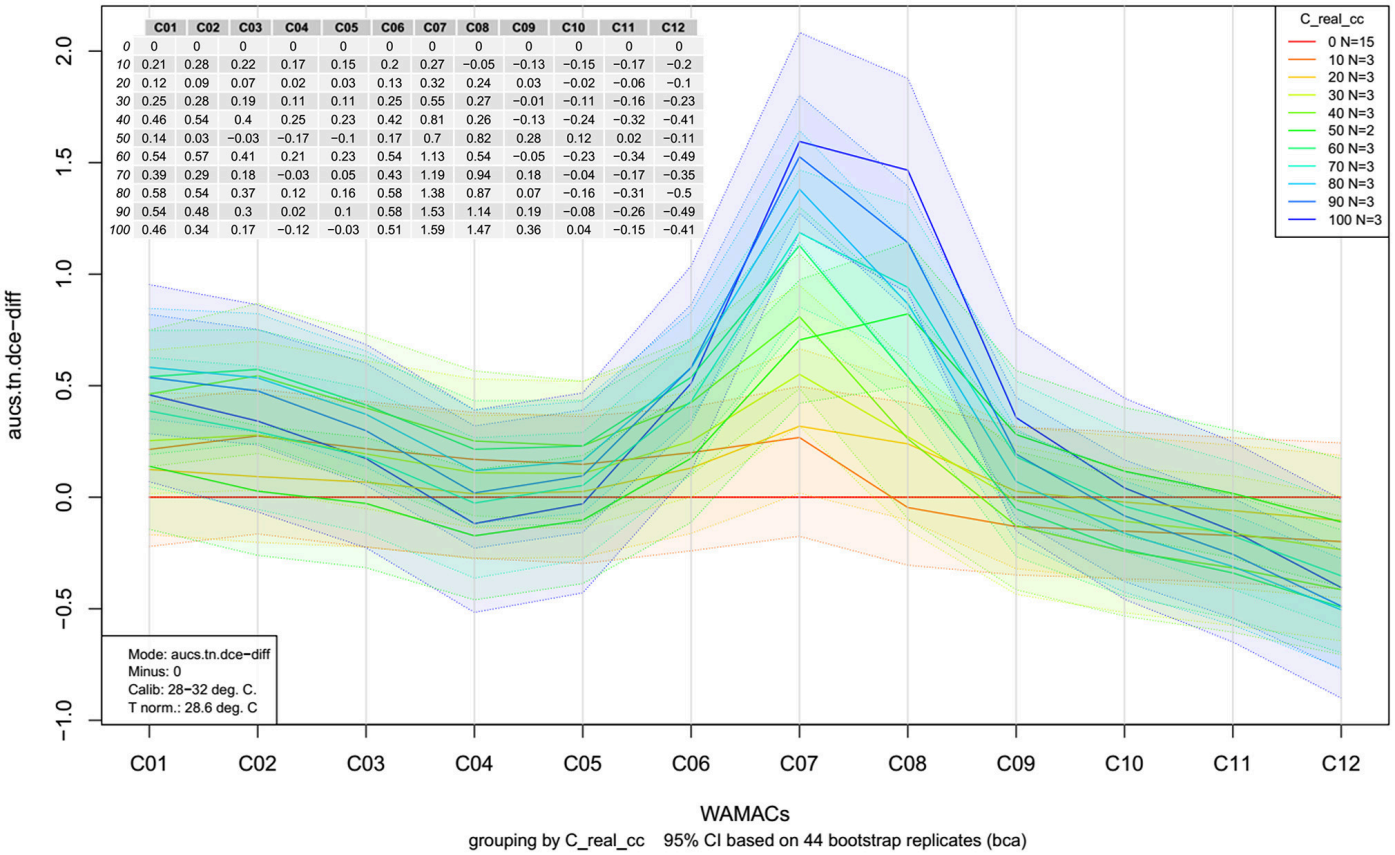
Aquagrams with 95% confidence intervals of Milli-Q water and aqueous solutions of potassium-chloride in the concentration range of 10–100 mM calculated on the MSC transformed absorbance (logT-1) spectra in the spectral range of 1,300–1,600 nm (OH first overtone) using the linearized version of the “temperature-based” mode with average values.

The temperature based aquagrams without and with confidence intervals for our dataset of aqueous solutions of potassium-chloride spectra are presented in Figures 13, 14, respectively. The linearized version of the temperature-based aquagram for Figure 14 is plotted in Figure 15, where the additional table shows average values at all WAMACs.

Further understanding can be obtained from the temperature-based aquagram. The addition of, for instance, 90 mM potassium-chloride to Milli-Q water results in structural changes equivalent to temperature changes of about 0.54, 0.48, 0.3, 0.02, 0.1, 0.58, 1.53, 1.14, 0.19, −0.08, −0.26 and −0.49°C at C_1_, C_2_, C_3_, C_4_, C_5_, C_6_, C_7_, C_8_, C_9_, C_10_, C_11_, and C_12_ coordinates, respectively. Furthermore, the differences are statistically significant for calculated confidence intervals, e.g., the above listed differences between pure Milli-Q and 90 mM aqueous solution of potassium-chloride was significant (*p* < 0.05) at the coordinates C_1_, C_2_, C_3_, C_6_, C_7_, C_8_, C_11_, and C_12_.

## Concluding remarks

In this paper, the fundamentals of the aquaphotomics approach to data analysis have been presented and discussed. A variety of applications illustrate the potential of aquaphotomics as a powerful new spectroscopic tool to study various aspects of aqueous and biological systems, which are of interest in the pharmaceutical and biomedical fields. The process of analysis illustrated by the application of aquaphotomics analysis on aqueous salt solutions was intended as guidance for certain steps of the analysis with the simplest experimental system, which anyone can easily reproduce. Together with the examples from sources of literature referenced throughout the text, this paper should provide the basis for independent experimental work in this field. The existing aquaphotomics literature shows the results which are probably only the tip of the iceberg of possible applications. With the explained methodology of aquaphotomics analysis presented herein, we hope that scientists and chemometricians will implement it in their fields and come up with new ideas of applications as well as new and more sophisticated mathematical tools to contribute to this growing field.

## Author contributions

ZK, BP, and RT designed and performed experiments. ZK performed data analysis. JM, ZK, and RT interpreted results and wrote the manuscript.

### Conflict of interest statement

The authors declare that the research was conducted in the absence of any commercial or financial relationships that could be construed as a potential conflict of interest.

## References

[B1] Agelet L. E. Hurburgh C. R. Jr (2010). A tutorial on near infrared spectroscopy and its calibration. Crit. Rev. Anal. Chem. 40, 246–260. 10.1080/10408347.2010.515468

[B2] Andrews N. L. MacLean A. G. Saunders J. E. Barnes J. A. Loock H. P. Saad M.. (2014). Quantification of different water species in acetone using a NIR-triple-wavelength fiber laser. Opt. Express 22, 19337–19347. 10.1364/OE.22.019337 25321018

[B3] Atanassova S. (2015). Near infrared spectroscopy and aquaphotomics for monitoring changes during yellow cheese ripening. Agric. Sci. Tech. 7, 269–272.

[B4] Atanassova S. Tsenkova R. Vasu R. M. Koleva M. Dimitrov M. (2009). Identification of mastitis pathogens in raw milk by near infrared spectroscopy and SIMCA classification method. Sci. Works Univ. Food Tech. Plovdiv 56, 567–572.

[B5] Barnes R. Dhanoa M. Lister S. (1993). Correction to the description of standard normal variate (SNV) and de-trend (DT) transformations in practical spectroscopy with applications in food and beverage analysis-−2nd edition. J. Near Infrared Spectrosc. 1, 185–186. 10.1255/jnirs.21

[B6] Barnes R. Dhanoa M. S. Lister S. J. (1989). Standard normal variate transformation and de-trending of near-infrared diffuse reflectance spectra. Appl. Spectrosc. 43, 772–777. 10.1366/0003702894202201

[B7] Barzaghi S. Cremonesi K. Cattaneo T. M. P. (2017). Influence of the presence of bioactive compounds in smart-packaging materials on water absorption using NIR spectroscopy and aquaphotomics. NIR News 28, 21–24. 10.1177/0960336017695731

[B8] Bázár G. Kovacs Z. Tanaka M. Furukawa A. Nagai A. Osawa M.. (2015). Water revealed as molecular mirror when measuring low concentrations of sugar with near infrared light. Anal. Chim. Acta 896, 52–62. 10.1016/j.aca.2015.09.014 26481987

[B9] Bázár G. Kovács Z. Tanaka M. Tsenkova R. (2014). Aquaphotomics and its extended water mirror concept explain why NIRS can measure low concentration aqueous solutions, in Aquaphotomics,“Understanding Water in Biological World”. The 5th Kobe University Brussels European Centre Symposium “Innovation, Environment, and Globalisation” (Brussels).

[B10] Bazar G. Kovacs Z. Tsenkova R. (2016). Evaluating spectral signals to identify spectral error. PLoS ONE 11:e0146249. 10.1371/journal.pone.0146249 26731541 PMC4701180

[B11] Bázár G. Romvári R. Szabó A Somogyi T. Éles V. Tsenkova R (2016). NIR detection of honey adulteration reveals differences in water spectral pattern. Food Chem. 194(Suppl. C), 873–880. 10.1016/j.foodchem.2015.08.092 26471630

[B12] Bro R. (1997). PARAFAC. Tutorial and applications. Chemometrics Intell. Lab. Sys. 38, 149–171. 10.1016/S0169-7439(97)00032-4

[B13] Buijs K. Choppin G. R. (1963). Near-infrared studies of the structure of water. I. pure water. J. Chem. Phys. 39, 2035–2041. 10.1063/1.1734579

[B14] Büning-Pfaue H. (2003). Analysis of water in food by near infrared spectroscopy. Food Chem. 82, 107–115. 10.1016/S0308-8146(02)00583-6

[B15] Cattaneo T. M. Vanoli M. Grassi M. Rizzolo A. Barzaghi S. (2016). The aquaphotomics approach as a tool for studying the influence of food coating materials on cheese and winter melon samples. J. Near Infrared Spectrosc. 24, 381–390. 10.1255/jnirs.1238

[B16] Cattaneo T. M. P. Cabassi G. Profaizer M. Giangiacomo R. (2009). Contribution of light scattering to near infrared absorption in milk. J. Near Infrared Spectrosc. 17, 337–343. 10.1255/jnirs.867

[B17] Cattaneo T. M. P. Stefania V. Elena N. Vittorio E. (2011). Influence of filtration processes on aqueous nanostructures by NIR spectroscopy. J. Chem. Chem. Eng. 5, 1046–1052.

[B18] Chandler D. (2002). Hydrophobicity: two faces of water. Nature 417:491. 10.1038/417491a 12037545

[B19] Chang K. Shinzawa H. Chung H. (2018). Concentration determination of inorganic acids that do not absorb near-infrared (NIR) radiation through recognizing perturbed NIR water bands by them and investigation of accuracy dependency on their acidities. Microchem. J. 139, 443–449. 10.1016/j.microc.2018.03.019

[B20] Chatani E. Tsuchisaka Y. Masuda Y. Tsenkova R. (2014). Water molecular system dynamics associated with amyloidogenic nucleation as revealed by real time near infrared spectroscopy and aquaphotomics. PLoS ONE 9:e101997. 10.1371/journal.pone.0101997 25013915 PMC4094474

[B21] Ciurczak E. W. Igne B. (2014). Pharmaceutical and Medical Applications of Near-Infrared Spectroscopy. Boca Raton, FL: CRC Press.

[B22] Cowe I. A. McNicol J. W. (1985). The use of principal components in the analysis of near-infrared spectra. Appl. Spectrosc. 39, 257–266. 10.1366/0003702854248944

[B23] Cui X. Cai W. Shao X. (2016). Glucose induced variation of water structure from temperature dependent near infrared spectra. RSC Adv. 6, 105729–105736. 10.1039/C6RA18912A

[B24] Cui X. Liu X. Yu X. Cai W. Shao X. (2017a). Water can be a probe for sensing glucose in aqueous solutions by temperature dependent near infrared spectra. Anal. Chim. Acta 957, 47–54. 10.1016/j.aca.2017.01.004 28107833

[B25] Cui X. Zhang J. Cai W. Shao X. (2017b). Chemometric algorithms for analyzing high dimensional temperature dependent near infrared spectra. Chemometr. Intell. Lab. Sys. 170, 109–117. 10.1016/j.chemolab.2017.08.010

[B26] Cupane A. Levantino M. Santangelo M. G. (2002). Near-infrared spectra of water confined in silica hydrogels in the temperature interval 365–5 K. J. Phys. Chem B 106, 11323–11328. 10.1021/jp026117m

[B27] Czarnecki M. A. Morisawa Y. Futami Y. Ozaki Y. (2015). Advances in molecular structure and interaction studies using near-infrared spectroscopy. Chem. Rev. 115, 9707–9744. 10.1021/cr500013u 26370249

[B28] Czarnik-Matusewicz B. Murayama K. Tsenkova R. Ozaki Y. (1999). Analysis of near-infrared spectra of complicated biological fluids by two-dimensional correlation spectroscopy: protein and fat concentration-dependent spectral changes of milk. Appl. Spectrosc. 53, 1582–1594. 10.1366/0003702991946046

[B29] Davison A. C. Hinkley D. V. (1997). Bootstrap methods and their applications, Cambridge Series in Statistical and Probabilistic Mathematics. Vol. 32 Cambridge, UK: Cambridge University Press.

[B30] Dhanoa M. Lister S. Sanderson R. Barnes R. (1994). The link between multiplicative scatter correction (MSC) and standard normal variate (SNV) transformations of NIR spectra. J. Near Infrared Spectrosc. 2, 43–47. 10.1255/jnirs.30

[B31] Doster W. Bachleitner A. Dunau R. Hiebl M. Lüscher E. (1986). Thermal properties of water in myoglobin crystals and solutions at subzero temperatures. Biophys. J. 50, 213–219. 10.1016/S0006-3495(86)83455-5 3741983 PMC1329738

[B32] Fornés V. Chaussidon J. (1978). An interpretation of the evolution with temperature of the ν2+ν3 combination band in water. J. Chem. Phys. 68, 4667–4671. 10.1063/1.435576

[B33] Giangiacomo R. Pani P. Barzaghi S. (2009). Sugars as a perturbation of the water matrix. J. Near Infrared Spectrosc. 17, 329–335. 10.1255/jnirs.861

[B34] Goto N. Bazar G. Kovacs Z. Kunisada M. Morita H. Kizaki S.. (2015). Detection of UV-induced cyclobutane pyrimidine dimers by near-infrared spectroscopy and aquaphotomics. Scient. Rep. 5:11808. 10.1038/srep11808 26133899 PMC4488872

[B35] Gowen A. (2012). Water and food quality. Contemp. Mater. 1, 31–37. 10.7251/COM1201031G

[B36] Gowen A. Tsenkova R. Esquerre C. Downey G. O'Donnell C. (2009a). Use of near infrared hyperspectral imaging to identify water matrix coordinates in mushrooms (Agaricus Bisporus) subjected to mechanical vibration. J. Near Infrared Spectrosc. 17, 363–371.

[B37] Gowen A. Tsuchisaka Y. O'Donnell C. Tsenkova R. (2011). Investigation of the potential of near infrared spectroscopy for the detection and quantification of pesticides in aqueous solution. Am. J. Anal. Chem. 2, 53–62. 10.4236/ajac.2011.228124

[B38] Gowen A. A. Amigo J. M. (2012). Applications of spectroscopy and chemical imaging in pharmaceutics, in Handbook of Biophotonics. Vol.3: Photonics in Pharmaceutics, Bionalysis and Environmental Research eds Popp J. Tuchin V. V. Chiou A. Heinemann S. (Weinheim: Wiley-VCH Verlag GmbHandCo), 71–88.

[B39] Gowen A. A. Amigo J. M. Tsenkova R. (2013). Characterisation of hydrogen bond perturbations in aqueous systems using aquaphotomics and multivariate curve resolution-alternating least squares. Anal. Chim. Acta 759, 8–20. 10.1016/j.aca.2012.10.007 23260672

[B40] Gowen A. A. Marini F. Tsuchisaka Y. De Luca S. Bevilacqua M. O'Donnell C.. (2015). On the feasibility of near infrared spectroscopy to detect contaminants in water using single salt solutions as model systems. Talanta 131, 609–618. 10.1016/j.talanta.2014.08.049 25281148

[B41] Gowen A. A. Tsenkova R. Esquerre C. Downey G. O'Donnell C. P. (2009b). Use of near infrared hyperspectral imaging to identify water matrix co-ordinates in mushrooms (Agaricus bisporus) subjected to mechanical vibration. J. Near Infrared Spectrosc. 17, 363–371. 10.1255/jnirs.860

[B42] Grant A. Davies A. M. C. Bilverstone T. (1989). Simultaneous determination of sodium hydroxide, sodium carbonate and sodium chloride concentrations in aqueous solutions by near-infrared spectrometry. Analyst 114, 819–822. 10.1039/an9891400819

[B43] Heiman A. Licht S. (1999). Fundamental baseline variations in aqueous near-infrared analysis. Anal. Chim. Acta 394, 135–147. 10.1016/S0003-2670(99)00312-8

[B44] Heise H. M. Winzen R. (2002). Chemometrics in Near-Infrared Spectroscopy, in Near-infrared spectroscopy: Principles, instruments and applications, eds Siesler H. W. Ozaki Y. Kawata S. Heise H. M. (Weinheim: Wiley-VCH Verlag GmbH), 125–162.

[B45] Hirschfeld T. (1985). Salinity determination using NIRA. Appl. Spectrosc. 39, 740–741. 10.1366/0003702854250293

[B46] Iwamoto M. Uozumi J. Nishinari K. (1987). Preliminary investigation of the state of water in foods by near infrared spectroscopy, in Proceedings of the International NIR/NIT Conference. eds Hollo J. Kafka K. J. Gonzcy J. (Budapest: Akademiai Kiado).

[B47] Jamrógiewicz M. (2012). Application of the near-infrared spectroscopy in the pharmaceutical technology. J. Pharm. Biomed. Anal. 66, 1–10. 10.1016/j.jpba.2012.03.009 22469433

[B48] Jinendra B. (2011). Near Infrared Spectroscopy and Aquaphotomics: Novel Tool for Biotic and Abiotic Stress Diagnosis of Soybean. Kobe: Kobe University.

[B49] Jinendra B. Tamaki K. Kuroki S. Vassileva M. Yoshida S. Tsenkova R. (2010). Near infrared spectroscopy and aquaphotomics: novel approach for rapid in vivo diagnosis of virus infected soybean. Biochem. Biophys. Res. Commun. 397, 685–690. 10.1016/j.bbrc.2010.06.007 20570650

[B50] Kang J. Cai W. Shao X. (2011). Quantitative determination by temperature dependent near-infrared spectra: a further study. Talanta 85, 420–424. 10.1016/j.talanta.2011.03.089 21645719

[B51] Kinoshita K. Kuze N. Kobayashi T. Miyakawa E. Narita H. Inoue-Murayama M.. (2016). Detection of urinary estrogen conjugates and creatinine using near infrared spectroscopy in Bornean orangutans (Pongo pygmaeus). Primates 57, 51–59. 10.1007/s10329-015-0501-3 26561334

[B52] Kinoshita K. Miyazaki M. Morita H. Vassileva M. Tang C. Li D. (2012). Spectral pattern of urinary water as a biomarker of estrus in the giant panda. Scientific reports 2. 10.1038/srep00856 PMC3504474 23181188

[B53] Kinoshita K. Morita H. Miyazaki M. Hama N. Kanemitsu H. Kawakami H. (2010). Near infrared spectroscopy of urine proves useful for estimating ovulation in giant panda (Ailuropoda melanoleuca). Analytical Methods 2, 1671–1675. 10.1039/c0ay00333f

[B54] Kojić D. Tsenkova R. Yasui M. (2017). Improving accuracy and reproducibility of vibrational spectra for diluted solutions. Anal. Chim. Acta 955, 86–97. 10.1016/j.aca.2016.12.019 28088284

[B55] Kojić D. Tsenkova R. Tomobe K. Yasuoka K. Yasui M. (2014). Water confined in the local field of ions. Chemphyschem 15, 4077–4086. 10.1002/cphc.201402381 25284338

[B56] Kovacs Z. Bázár G. Oshima M. Shigeoka S. Tanaka M. Furukawa A.. (2016). Water spectral pattern as holistic marker for water quality monitoring. Talanta 147, 598–608. 10.1016/j.talanta.2015.10.024 26592651

[B57] Liu Y. Ozaki Y. Noda I. (1996). Two-dimensional Fourier-transform near-infrared correlation spectroscopy study of dissociation of hydrogen-bonded N-methylacetamide in the pure liquid state. J. Phys. Chem. 100, 7326–7332. 10.1021/jp9534186

[B58] Luck W. A. (ed.). (1974). Structure of Water and Aqueous Solutions. Weinheim: Verlag Chemie.

[B59] Luck W. A. P. (1998). The importance of cooperativity for the properties of liquid water. J. Mol. Struct. 448, 131–142. 10.1016/S0022-2860(98)00343-3

[B60] Maeda H. Ozaki Y. Tanaka M. Hayashi N. Kojima T. (1995). Near Infrared spectroscopy and chemometrics studies of temperature-dependent spectral variations of water: relationship between spectral changes and hydrogen bonds. J. Near Infrared Spectrosc. 3, 191–201. 10.1255/jnirs.69

[B61] Manley M. (2014). Near-infrared spectroscopy and hyperspectral imaging: non-destructive analysis of biological materials. Chem. Soc. Rev. 43, 8200–8214. 10.1039/C4CS00062E 25156745

[B62] Martens H. Martens M. (2001). Multivariate analysis of quality. An introduction. (Chichester: Wiley).

[B63] Matija L. Muncan J. Mileusnic I. Koruga D. (2017). Fibonacci nanostructures for novel nanotherapeutical approach, in Nano-and Microscale Drug Delivery, S.ystems. Elsevier, 49–74. 10.1016/B978-0-323-52727-9.00004-2

[B64] Matija L. Tsenkova R. Miyazaki M. Banba K. Muncan J. (2012). Aquagrams: Water spectral pattern as characterization of hydrogenated nanomaterial. FME Transac. 40, 51–56.

[B65] Matija L. Tsenkova R. Munćan J. Miyazaki M. Banba K. Tomić M. (2013). Fullerene based nanomaterials for biomedical applications: engineering, functionalization and characterization, in: Advanced Materials Research: Trans Tech Publ, 224–238. 10.4028/www.scientific.net/AMR.633.224

[B66] Meilina H. Kuroki S. Jinendra B. M. Ikuta K. Tsenkova R. (2009). Double threshold method for mastitis diagnosis based on NIR spectra of raw milk and chemometrics. Biosyst. Eng. 104, 243–249. 10.1016/j.biosystemseng.2009.04.006

[B67] Meilina H. Putra A. Tsenkova R. (2011). Frequency of use minute concentrations of cadmium in aqueous solution by near infrared spectroscopy and aquaphotomics, in Proceedings of the Annual International Conference, Syiah Kuala University-Life Sciences & Engineering Chapter.

[B68] Monakhova Y. B. Pozharov M. V. Zakharova T. V. Khvorostova E. K. Markin A. V. Lachenmeier D. W. (2014). Association/Hydrogen bonding of acetone in polar and non-polar solvents: NMR and NIR spectroscopic investigations with chemometrics. J. Solution Chem. 43, 1963–1980. 10.1007/s10953-014-0249-1

[B69] Munćan J. Matija L. Simić-Krstić J Nijemčević S Koruga D. (2014). Discrimination of mineral waters using near infrared spectroscopy and aquaphotomics. Hemijska industrija 68, 257–264. 10.2298/HEMIND130412049M

[B70] Munćan J. Mileusnić I Matović V Šakota Rosić J Matija L. (2016a). The prospects of aquaphotomics in biomedical science and engineering, in Aquaphotomics: Understanding Water in Biology – 2nd International Symposium. (Kobe University, Kobe, Japan).

[B71] Munćan J. Mileusnić I Šakota Rosić J Vasić-Milovanović A Matija L. (2016b). Water properties of soft contact lenses: a comparative near-infrared study of two hydrogel materials. Int. J. Polym. Sci. 1–8. 10.1155/2016/3737916

[B72] Murayama K. Czarnik-Matusewicz B. Wu Y. Tsenkova R. Ozaki Y. (2000). Comparison between conventional spectral analysis methods, chemometrics, and two-dimensional correlation spectroscopy in the analysis of near-infrared spectra of protein. Appl. Spectrosc. 54, 978–985. 10.1366/0003702001950715

[B73] Murayama K. Yamada K. Tsenkova R. Wang Y. Ozaki Y. (1998). Near-infrared spectra of serum albumin and γ-globulin and determination of their concentrations in phosphate buffer solutions by partial least squares regression. Vib. Spectrosc. 18, 33–40. 10.1016/S0924-2031(98)00034-4

[B74] Næs T. Isaksson T. Fearn T. Davies T. A. (2002). User Friendly Guide to Multivariate Calibration and Classification. (Chichester: NIR publications).

[B75] Nakakimura Y. Vassileva M. Stoyanchev T. Nakai K. Osawa R. Kawano J. (2012). Extracellular metabolites play a dominant role in near-infrared spectroscopic quantification of bacteria at food-safety level concentrations. Anal. Methods 4, 1389–1394. 10.1039/c2ay05771a

[B76] Noda I. Liu Y. Ozaki Y. Czarnecki M. A. (1995). Two-dimensional Fourier transform near-infrared correlation spectroscopy studies of temperature-dependent spectral variations of oleyl alcohol. J. Phys. Chem. 99, 3068–3073. 10.1021/j100010a016

[B77] Noda I. Ozaki Y. (eds.). (2004). Two-Dimensional Correlation Spectroscopy – Applications in Vibrational and Optical Spectroscopy. Chichester: John Wiley and Sons Inc.

[B78] Norris K. H. Williams P. C. (1984). Optimization of mathematical treatments of raw near-infrared signal in the. measurement of protein in hard red spring wheat. i. influence of particle size. Cereal Chem. 61, 158–165.

[B79] Omar A. F. Atan H. Matjafri M. Z. (2012). NIR spectroscopic properties of aqueous acids solutions. Molecules 17, 7440–7450. 10.3390/molecules17067440 22706373 PMC6268505

[B80] Osborne B. G. Fearn T. Hindle P. H. Practical N. I. R. (1993). Spectroscopy with Applications in Food and Beverage Analysis. Harlow: Longman scientific and technical.

[B81] Ozaki Y. (2002). Applications in Chemistry, in Near-infrared Spectroscopy: Principles, Instruments, Applications, eds Siesler H. W. Ozaki Y. Kawata S. Heise H. M. (Weinheim: Verlag GmbH), 179–212.

[B82] Ozaki Y. Katsumoto Y. Jiang J.-H. Liang Y. (2003). Spectral analysis in the, NIR region in Useful Advanced Information in the Field of near Infrared Spectroscopy, ed Tsuchikawa S. (Trivandrum: Research Signpost), 307.

[B83] Pasquini C. (2018). Near infrared spectroscopy: a mature analytical technique with new perspectives – A review. Anal. Chim. Acta. 1026, 8–36. 10.1016/j.aca.2018.04.004 29852997

[B84] Patil R. (2015). Noise reduction using wavelet transform and singular vector decomposition. Procedia Comput. Sci. 54, 849–853. 10.1016/j.procs.2015.06.099

[B85] Peinado A. C. van den Berg F. Blanco M. Bro R. (2006). Temperature-induced variation for NIR tensor-based calibration. Chemometr. Intell. Lab. Sys. 83, 75–82. 10.1016/j.chemolab.2006.01.006

[B86] Pollner B. Kovacs Z. (2016). Multivariate data analysis tools for, R including aquaphotomics methods, aquap2

[B87] Putra A. Faridah F. Inokuma E. Santo R. (2010). Robust spectral model for low metal concentration measurement in aqueous solution reveals the importance of water absorbance bands. J. Sains dan Teknologi Reaksi 210:8.

[B88] Putra A. Vassileva M. Santo R. Tsenkova R. (2017). An efficient near infrared spectroscopy based on aquaphotomics technique for rapid determining the level of Cadmium in aqueous solution, in IOP Conference Series: Materials Science and Engineering (Kuala Lumpur).

[B89] R Core Team (2017). A language and Environment for Statistical Computing. Vienna: R Foundation for Statistical Computing.

[B90] Reeves J. B. (1995). Efforts to quantify changes in near-infrared spectra caused by the influence of water, pH, ionic strength, and differences in physical state. Appl. Spectrosc. 49, 181–187. 10.1366/0003702953963788

[B91] Remagni M. C. Morita H. Koshiba H. Cattaneo T. M. P. Tsenkova R. (2013). Near infrared spectroscopy and aquaphotomics as tools for bacteria classification. NIR2013, in Proceedings: Picking Up Good Vibrations (La Grande-Motte), 602.

[B92] Rinnan Å. van den Berg F. Engelsen S. B. (2009). Review of the most common pre-processing techniques for near-infrared spectra. TrAC Trends Anal. Chem. 28, 1201–1222. 10.1016/j.trac.2009.07.007

[B93] Robertson W. H. Diken E. G. Price E. A. Shin J.-W. Johnson M. A. (2003). Spectroscopic determination of the OH– solvation shell in the OH–·(H2O) n clusters. Science 299, 1367–1372. 10.1126/science.1080695 12543981

[B94] Roggo Y. Chalus P. Maurer L. Lema-Martinez C. Edmond A. Jent N. (2007). A review of near infrared spectroscopy and chemometrics in pharmaceutical technologies. J. Pharm. Biomed. Anal. 44, 683–700. 10.1016/j.jpba.2007.03.023 17482417

[B95] Šakota Rosić J. Munćan J. Mileusnić I Kosić B Matija L. (2016). Detection of protein deposits using NIR spectroscopy. Soft Mater. 14, 264–271. 10.1080/1539445X.2016.1198377

[B96] Sakudo A. Tsenkova R. Onozuka T. Morita K. Li S. Warachit J.. (2005). A novel diagnostic method for human immunodeficiency virus Type-1 in plasma by near-infrared spectroscopy. Microbiol. Immunol. 49, 695–701. 10.1111/j.1348-0421.2005.tb03648.x 16034213

[B97] Sakudo A. Tsenkova R. Tei K. Morita H. Ikuta K. Onodera T. (2006a). *Ex vivo* tissue discrimination by visible and near-infrared spectra with chemometrics. J. Vet. Med. Sci. 68, 1375–1378. 10.1292/jvms.68.1375 17213714

[B98] Sakudo A. Tsenkova R. Tei K. Onozuka T. Ikuta K. Yoshimura E.. (2006b). Comparison of the vibration mode of metals in HNO3 by a partial least-squares regression analysis of near-infrared spectra. Biosci. Biotechnol. Biochem. 70, 1578–1583. 10.1271/bbb.50619 16861790

[B99] Sakudo A. Yoshimura E. Tsenkova R. Ikuta K. Onodera T. (2007). Native state of metals in non-digested tissues by partial least squares regression analysis of visible and near-infrared spectra. J. Toxicol. Sci. 32, 135–141. 10.2131/jts.32.135 17538238

[B100] Sartor G. Hallbrucker A. Mayer E. (1995). Characterizing the secondary hydration shell on hydrated myoglobin, hemoglobin, and lysozyme powders by its vitrification behavior on cooling and its calorimetric glass–>liquid transition and crystallization behavior on reheating. Biophys. J. 69, 2679–2694. 8599674 10.1016/S0006-3495(95)80139-6 PMC1236505

[B101] Šašić S. Segtnan V. H. Ozaki Y (2002). Self-modeling curve resolution study of temperature-dependent near-infrared spectra of water and the investigation of water structure. J. Phys. Chem. A 106, 760–766. 10.1021/jp013436p

[B102] Savitzky A. Golay M. J. (1964). Smoothing and differentiation of data by simplified least squares procedures. Anal. Chem. 36, 1627–1639. 10.1021/ac60214a047 22324618

[B103] Segtnan V. H. Sasić S Isaksson T Ozaki Y. (2001). Studies on the structure of water using two-dimensional near-infrared correlation spectroscopy and principal component analysis. Anal. Chem. 73, 3153–3161. 10.1021/ac010102n 11467567

[B104] Shan R. Zhao Y. Fan M. Liu X. Cai W. Shao X. (2015). Multilevel analysis of temperature dependent near-infrared spectra. Talanta 131, 170–174. 10.1016/j.talanta.2014.07.081 25281089

[B105] Shao X. Cui X. Yu X. Cai W. (2018). Mutual factor analysis for quantitative analysis by temperature dependent near infrared spectra. Talanta 183, 142–148. 10.1016/j.talanta.2018.02.043 29567156

[B106] Shao X. Kang J. Cai W. (2010). Quantitative determination by temperature dependent near-infrared spectra. Talanta 82, 1017–1021. 10.1016/j.talanta.2010.06.009 20678661

[B107] Shao X. G. Leung A. K. Chau F. T. (2003). Wavelet: a new trend in chemistry. Acc. Chem. Res. 36, 276–283. 10.1021/ar990163w 12693925

[B108] Siesler H. W. Ozaki Y. Kawata S. Heise H. M. (2008). Near-Infrared Spectroscopy: Principles, Instruments, Applications. Weinheim: John, Wiley and Sons.

[B109] Slavchev A. Kovacs Z. Koshiba H. Bazar G. Pollner B. Krastanov A. (2017). Monitoring of water spectral patterns of lactobacilli development as a tool for rapid selection of probiotic candidates. J. Near Infrared Spectrosc. 25:0967033517741133 10.1177/0967033517741133

[B110] Slavchev A. Kovacs Z. Koshiba H. Nagai A. Bázár G. Krastanov A.. (2015). Monitoring of water spectral pattern reveals differences in probiotics growth when used for rapid bacteria selection. PLoS ONE 10:e0130698. 10.1371/journal.pone.0130698 26133176 PMC4489812

[B111] Smith J. D. Cappa C. D. Wilson K. R. Cohen R. C. Geissler P. L. Saykally R. J. (2005). Unified description of temperature-dependent hydrogen-bond rearrangements in liquid water. Proc. Natl. Acad. Sci. U.S.A. 102, 14171–14174. 10.1073/pnas.0506899102 16179387 PMC1242322

[B112] Steen G. W. Fuchs E. C. Wexler A. D. Offerhaus H. L. (2015). Identification and quantification of 16 inorganic ions in water by Gaussian curve fitting of near-infrared difference absorbance spectra. Appl. Opt. 54, 5937–5942. 10.1364/AO.54.005937 26193135

[B113] Takemura G. Bázár G. Ikuta K. Yamaguchi E. Ishikawa S. Furukawa A.. (2015). Aquagrams of raw milk for oestrus detection in dairy cows. Reprod. Domest. Anim. 50, 522–525. 10.1111/rda.12504 25704193

[B114] Tillmann P. Paul C. (1998). The repeatability file—a tool for reducing the sensitivity of near infrared spectroscopy calibrations to moisture variation. J. Near Infrared Spectrosc. 6, 61–68. 10.1255/jnirs.122

[B115] Timmerman M. E. (2006). Multilevel component analysis. Br. J. Math. Stat. Psychol. 59, 301–320. 10.1348/000711005X67599 17067414

[B116] Tsenkova R. (2004). Near Infrared Spectroscopy of Raw Milk for Cow's Biomonitoring. Ph.D. thesis, Hokkaido University (?????).

[B117] Tsenkova R. (2005). Visible-near infrared perturbation spectroscopy: Water in action seen as a source of information, in 12th International Conference on Near-infrared Spectroscopy (Auckland), 607–612.

[B118] Tsenkova R. (2006a). Aquaphotomics. Aquaphotomics and chambersburg. NIR News 17, 12–10. 10.1255/nirn.916

[B119] Tsenkova R. (2006b). Aquaphotomics: exploring water-light interactions for a better understanding of the biological world. Part 2: Japanese food, language and why NIR for diagnosis? NIR News 17, 8–14. 10.1255/nirn.904

[B120] Tsenkova R. (2006c). AquaPhotomics: water absorbance pattern as a biological marker. NIR News 17, 13–10. 10.1255/nirn.1014

[B121] Tsenkova R. (2007). AquaPhotomics: water absorbance pattern as a biological marker for disease diagnosis and disease understanding. NIR News 18, 14–16. 10.1255/nirn.1014

[B122] Tsenkova R. (2008a). Aquaphotomics: acquiring spectra of various biological fluids of the same organism reveals the importance of water matrix absorbance coordinates and the aquaphotome for understanding biological phenomena. NIR News 19, 13–15.

[B123] Tsenkova R. (2008b). Aquaphotomics: the extended water mirror effect explains why small concentrations of protein in solution can be measured with near infrared light. NIR News 19, 13–14.

[B124] Tsenkova R. (2008c). Aquaphotomics: VIS-near infrared spectrum of water as biological marker, in Conference on the Physics, Chemistry and Biology of Water (Sofia).

[B125] Tsenkova R. (2009). Aquaphotomics: dynamic spectroscopy of aqueous and biological systems describes peculiarities of water. J. Near Infrared Spectrosc. 17, 303–313. 10.1255/jnirs.869

[B126] Tsenkova R. (2010). Aquaphotomics: water in the biological and aqueous world scrutinised with invisible light. Spectrosc. Eur. 22, 6–10.

[B127] Tsenkova R. Atanassova S. (2002). Mastitis diagnostics by near infrared spectra of cow's milk, blood and urine using soft independent modelling of class analogy classification, in Near Infrared Spectroscopy: Proceedings of the 10th International Conference, eds Davies A. M. C. Cho R. K. (Chichester: NIR Publications).

[B128] Tsenkova R. Atanassova S. Kawano S. Toyoda K. (2001a). Somatic cell count determination in cow's milk by near-infrared spectroscopy: a new diagnostic tool. J. Anim. Sci. 79, 2550–2557. 10.2527/2001.79102550x 11721833

[B129] Tsenkova R. Atanassova S. Ozaki Y. Toyoda K. Itoh K. (2001b). Near-infrared spectroscopy for biomonitoring: influence of somatic cell count on cow's milk composition analysis. Intl. Dairy J. 11, 779–783. 10.1016/S0958-6946(01)00110-8

[B130] Tsenkova R. Atanassova S. Toyoda K. (2001c). Near infrared spectroscopy for diagnosis: influence of mammary gland inflammation on cow' s milk composition measurement. Near Infrared Anal. 2, 59–66. 10.11357/jsam1937.61

[B131] Tsenkova R. Fockenberg C. Koseva N. Sakudo A. Parker M. (2007a). Aquaphotomics: water absorbance patterns in NIR range used for detection of metal ions reveal the importance of sample preparation, in 13th International Conference on Near Infrared spectroscopy (Umea), 03–02.

[B132] Tsenkova R. Iso E. Parker M. Fockenberg C. Okubo M. (2007b). Aquaphotomics: a NIRS investigation into the perturbation of water spectrum in an aqueous suspension of mesoscopic scale polystyrene spheres, in 13th International Conference on Near Infrared Spectroscopy (Umea), A–04.

[B133] Tsenkova R. Kovacs Z. Kubota Y. (2015). Aquaphotomics: near infrared spectroscopy water states in biological systems, in Membrane Hydration ed Anibal Disalvo E. (Berlin: Springer), 189–211. 10.1007/978-3-319-19060-0_8 26438266

[B134] Tsenkova R. Morita H. Shinzawa H. Hogeveen H. Hillerton J. E. Ikuta K. (2005). Near infrared spectroscopy for cow identification and *in-vivo* mastitis diagnosis, in Mastitis in Dairy Production. Current Knowledge and Future Solutions, 4th IDF International Mastitis Conference (Maastricht), 901.

[B135] Tsenkova R. N. (1994). Near-infrared spectroscopy of individual cow milk as a means for automated monitoring of udder health and milk quality, in Proceedings of Third International Dairy Housing Conference (Orlando, FL).

[B136] Tsenkova R. N. Iordanova I. K. Toyoda K. Brown D. R. (2004). Prion protein fate governed by metal binding. Biochem. Biophys. Res. Commun. 325, 1005–1012. 10.1016/j.bbrc.2004.10.135 15541389

[B137] Wang Y. Murayama K. Myojo Y. Tsenkova R. Hayashi N. Ozaki Y. (1998). Two-dimensional fourier transform near-infrared spectroscopy study of heat denaturation of ovalbumin in aqueous solutions. J. Phys. Chem. B 102, 6655–6662. 10.1021/jp9816115

[B138] Weber J. M. Kelley J. A. Nielsen S. B. Ayotte P. Johnson M. A. (2000). Isolating the spectroscopic signature of a hydration shell with the use of clusters: superoxide tetrahydrate. Science 287, 2461–2463. 10.1126/science.287.5462.2461 10741960

[B139] Weber J. M. Kelley J. A. Robertson W. H. Johnson M. A. (2001). Hydration of a structured excess charge distribution: infrared spectroscopy of the ${\text{O}}_{2}^{-}$·(H_2_O)_*n*_, (1 ≤ n ≤ 5) clusters. J. Chem. Phys. 114, 2698–2706. 10.1063/1.1338529

[B140] Wenz J. J. (2018). Examining water in model membranes by near infrared spectroscopy and multivariate analysis. Biochim. Biophys. Acta Biomembr. 1860, 673–682. 10.1016/j.bbamem.2017.12.007 29229525

[B141] Williams P. Norris K. (1987). Near-Infrared Technology in the Agricultural and Food Industries. St. Paul, MI: American Association of Cereal Chemists Inc.

[B142] Wold S. Geladi P. Esbensen K. Öhman J. (1987). Multi-way principal components-and PLS-analysis. J. Chemom. 1, 41–56. 10.1002/cem.1180010107

[B143] Wold S. Sjöström M. (1977). SIMCA: a method for analyzing chemical data in terms of similarity and analogy, in Chemometrics: Theory and Application, ed Kowalski B. R. (Washington DC: American Chemical Society), 243–282.

[B144] Workman J. Jr. (2000). The Handbook of Organic Compounds: NIR, IR, Raman, and, UV-, VIS Spectra Featuring Polymers and Surfactants. London: Elsevier.

[B145] Workman J. (2016). The Concise Handbook of Analytical Spectroscopy, Vol. 3 Singapore: World Scientific Publishing Co. Pte. Ltd.

[B146] Wu H. L. Shibukawa M. Oguma K. (1998). An alternating trilinear decomposition algorithm with application to calibration of HPLC–DAD for simultaneous determination of overlapped chlorinated aromatic hydrocarbons. J. Chemometr. Soc. 12, 1–26.

[B147] Xantheas S. S. (1995). *Ab initio* studies of cyclic water clusters (H_2_O)_*n*_, *n* = 1–6. III. Comparison of density functional with MP2 results. J. Chem. Phys. 102, 4505–4517. 10.1063/1.469499

[B148] Yuan B. Murayama K. Wu Y. Tsenkova R. Dou X. Era S.. (2003). Temperature-dependent near-infrared spectra of bovine serum albumin in aqueous solutions: spectral analysis by principal component analysis and evolving factor analysis. Appl. Spectrosc. 57, 1223–1229. 10.1366/000370203769699072 14639749

